# The Complete Genome Sequence of the Plant Growth-Promoting Bacterium *Pseudomonas* sp. UW4

**DOI:** 10.1371/journal.pone.0058640

**Published:** 2013-03-13

**Authors:** Jin Duan, Wei Jiang, Zhenyu Cheng, John J. Heikkila, Bernard R. Glick

**Affiliations:** Department of Biology, University of Waterloo, Waterloo, Ontario, Canada; Wageningen University, Netherlands

## Abstract

The plant growth-promoting bacterium (PGPB) *Pseudomonas* sp. UW4, previously isolated from the rhizosphere of common reeds growing on the campus of the University of Waterloo, promotes plant growth in the presence of different environmental stresses, such as flooding, high concentrations of salt, cold, heavy metals, drought and phytopathogens. In this work, the genome sequence of UW4 was obtained by pyrosequencing and the gaps between the contigs were closed by directed PCR. The *P.* sp. UW4 genome contains a single circular chromosome that is 6,183,388 bp with a 60.05% G+C content. The bacterial genome contains 5,423 predicted protein-coding sequences that occupy 87.2% of the genome. Nineteen genomic islands (GIs) were predicted and thirty one complete putative insertion sequences were identified. Genes potentially involved in plant growth promotion such as indole-3-acetic acid (IAA) biosynthesis, trehalose production, siderophore production, acetoin synthesis, and phosphate solubilization were determined. Moreover, genes that contribute to the environmental fitness of UW4 were also observed including genes responsible for heavy metal resistance such as nickel, copper, cadmium, zinc, molybdate, cobalt, arsenate, and chromate. Whole-genome comparison with other completely sequenced *Pseudomonas* strains and phylogeny of four concatenated “housekeeping” genes (16S rRNA, *gyrB*, *rpoB* and *rpoD*) of 128 *Pseudomonas* strains revealed that UW4 belongs to the *fluorescens* group, *jessenii* subgroup.

## Introduction

*Pseudomonas* is one of the most diverse and prevalent genera that are present in all natural environments. *P.* sp. UW4 is a well-studied PGPB that was isolated from the rhizosphere of reeds in Waterloo, Ontario [1]. This strain has the ability to utilize 1-aminocyclopropane-1-carboxylate (ACC) as a sole source of nitrogen and promote canola seedling root elongation in growth pouches under gnotobiotic conditions [1].

Strain UW4 was originally designated *Pseudomonas* sp. on the basis of growth on *Pseudomonas* Agar F (PAF) selective medium and siderophore production. Subsequently, the bacterium was designated as *Enterobacter cloacae* UW4 based on the results of fatty acid analysis [2]. However, after sequencing a partial 16S ribosomal RNA gene from UW4, the data indicated that this strain is *Pseudomonas putida* [3], and the genus and species were further confirmed by thorough detailed metabolic profiling (MicroLog System, Release 4.0).

In 1998, the gene encoding ACC deaminase was isolated from UW4. When the ACC deaminase gene of UW4, and its upstream DNA sequence, was introduced into *Escherichia coli* DH5α, *P. putida* ATCC 17399 or *Pseudomonas fluorescens* ATCC 17400, the gene was expressed and the transformants were able to promote canola seedling root elongation [2]. Furthermore, when the *acdS* gene in UW4 was disrupted, the strain lost its capability to promote root elongation [4]. The gene upstream of *acdS* and the intergenic region between the two genes are involved in a complex mode of transcriptional regulation [5]–[7].

Subsequently, a number of studies focused on the impact of the *acdS* gene of UW4 on plant growth in the presence of different environmental stresses. For example, when the *acdS* gene and its regulatory region was introduced into a biocontrol strain, *P. fluorescens* strain CHA0, the transformed strain showed improved ability to protect cucumber against *Pythium* damping-off, and potato tubers against *Erwinia* soft rot [8]. Furthermore, transgenic tomato plants expressing the UW4 ACC deaminase showed reduced symptoms of *Verticillium* wilt [9]. In the presence of heavy metals including Cd, Co, Cu, Mg, Ni, Pb, and Zn, ACC deaminase-producing tomato and canola plants showed less deleterious effects of the metals on plant growth compared to the non-transgenic plants [10]–[12]. In another study, under flood conditions, tomato plants inoculated with UW4’s *acdS*-containing bacterial strains showed a significant tolerance to flooding stress [13]. In addition, UW4 has been shown to enhance plant growth in the presence of flooding [14], heavy metals [15], cold [16], high concentrations of salt [16], and phytopathogens [17]–[18].

In an effort to better understand the interaction between plants and free-living PGPB, the proteomes of wild type UW4 and its *acdS* minus mutant were investigated upon treatment with canola root exudates [19]. Furthermore, when UW4 was exposed to 2 mM Ni, bacterial proteins involved in heavy metal detoxification such as stress adaptation, anti-oxidative stress, and heavy metal efflux proteins were up-regulated significantly [20]. More recently, Cheng et al. [21] analyzed the protein expression profile of canola plants inoculated with UW4 or its *acdS* minus mutant under salinity stress. As expected, many of the differentially expressed proteins in the plants are related to salt stress tolerance. Moreover, it was observed that the enzyme ACC deaminase played an important role in the salt response of canola plants. For example, the expression of proteins involved in photosynthesis decreased to a lesser extent if the plants were treated with wild type UW4 prior to salt exposure, and the plants were healthier due to the lowered stress ethylene levels [21].

In 2009, a proteome reference map of UW4 was published [22], representing 275 different UW4 proteins. Although this map represents only ∼5% of the total number of proteins synthesized by UW4, it should facilitate future proteomic studies with this bacterium.

Here, we report the complete genomic sequence of UW4. Based on the phylogeny of whole genome and concatenated four “housekeeping” genes, the name of the organism has been changed back to *P.* sp. UW4. Knowing the complete genome sequence of UW4 will help unravel the complex biological mechanisms that UW4 uses to promote plant growth. The genome analyses will provide a fundamental basis for future studies towards fully understanding the functioning of this organism. Furthermore, comparisons among the completely sequenced *Pseudomonas* genomes will help to determine the pan and core-*Pseudomonas* genome, and offer insights into evolutionary changes between *Pseudomonas* spp.

## Results and Discussion

### General Genome Features

**Figure 1:**
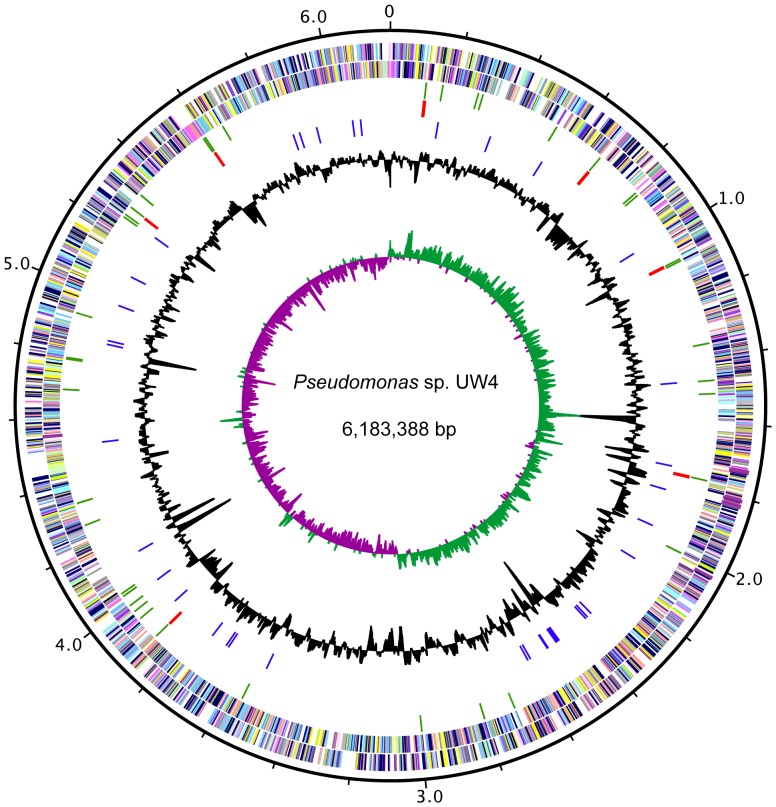
Circular genome map of *P.* sp. UW4. From the outside in, the outer black circle shows the scale line in Mbps; circles 2 and 3 represent the coding region with the colors of the COG categories; circle 4 and 5 show tRNA (green) and rRNA (red), respectively; circle 6 displays the IS elements (blue); circle 7 shows the genomic islands (orange); circle 8 represents mean centered G+C content (bars facing outside-above mean, bars facing inside-below mean); circle 9 shows GC skew (G−C)/(G+C). GC content and GC skew were calculated using a 10-kb window in steps of 200 bp.

The genome of *P.* sp. UW4 has a single circular chromosome of 6,183,388 bp (Figure 1) and an average G+C content of 60.05% (Table 1). The genome contains 5,423 predicted CDSs with an average length of 995 bp. Among these CDSs, 4378 (80.7%) genes could be classified into COG families composed of 22 categories (Table 2). Twenty genes were assigned pseudogenes due to missing an N- and/or C-terminus, or frameshift mutation (Table S1). Coding regions cover 87.2% of the whole genome. Biological roles were assigned to 4,158 (76.7%) genes of the predicted coding sequences based on similarity searches and experimental evidence. The remaining coding sequences were classified as proteins with unknown function. Among the 1265 (23.3%) CDSs with unknown function, 132 hypothetical proteins had no identifiable counterpart when searched against protein databases using a cutoff *E* value of 10^−5^, indicating putative unique genes present in UW4 that have not yet been reported for other organisms. A total of seven rRNA operons including eight 5S rRNAs, seven 16S rRNAs, and seven 23S rRNAs are present on the chromosome. In addition, 72 tRNA genes that represent all 20 amino acids, and a tRNA for selenocysteine, were identified.

**Table 1:** General Features of *P.* sp. UW4 Genome.

Features	Chromosome
Size (bp)	6,183,388
G+C content (%)	60.05
Number of CDSs	5423
Total CDSs size (bp)	5,397,636
Coding %	87.2
Average CDS length (nt)	995
Pseudogenes	20
tRNAs	72
rRNA genes (clusters)	22 (7)
Number of genes with assigned function	4158 (76.7%)
Number of genes without assigned function	1265 (23.3%)
Number of predicted enzymes	1548 (28.5%)

**Table 2:** COG Functional Categories of *P.* sp. UW4.

	Functional Category	UW4
A	RNA processing and modification	1
B	Chromatin structure and dynamics	3
C	Energy production and conversion	288
D	Cell cycle control, cell division, chromosome partitioning	37
E	Amino acid transport and metabolism	500
F	Nucleotide transport and metabolism	90
G	Carbohydrate transport and metabolism	232
H	Coenzyme transport and metabolism	156
I	Lipid transport and metabolism	255
J	Translation, ribosomal structure and biogenesis	174
K	Transcription	431
L	Replication, recombination and repair	175
M	Cell wall/membrane/envelope biogenesis	261
N	Cell motility	114
O	Posttranslational modification, protein turnover, chaperones	179
P	Inorganic ion transport and metabolism	229
Q	Secondary metabolites biosynthesis, transport and catabolism	102
R	General function prediction only	441
S	Function unknown	374
T	Signal transduction mechanisms	231
U	Intracellular trafficking, secretion, and vesicular transport	43
V	Defense mechanisms	62
Total		4378

The chromosome of *P.* sp. UW4 displays two clear GC skew transitions, which corresponds with its *oriC* and terminus (Figure 1). The *oriC* site contains nine conserved DnaA-binding boxes (TTATCCACA and closely related sequences) [23]–[24] and is located between the *rpmH* and the *dnaA* genes.

**Figure 2:**
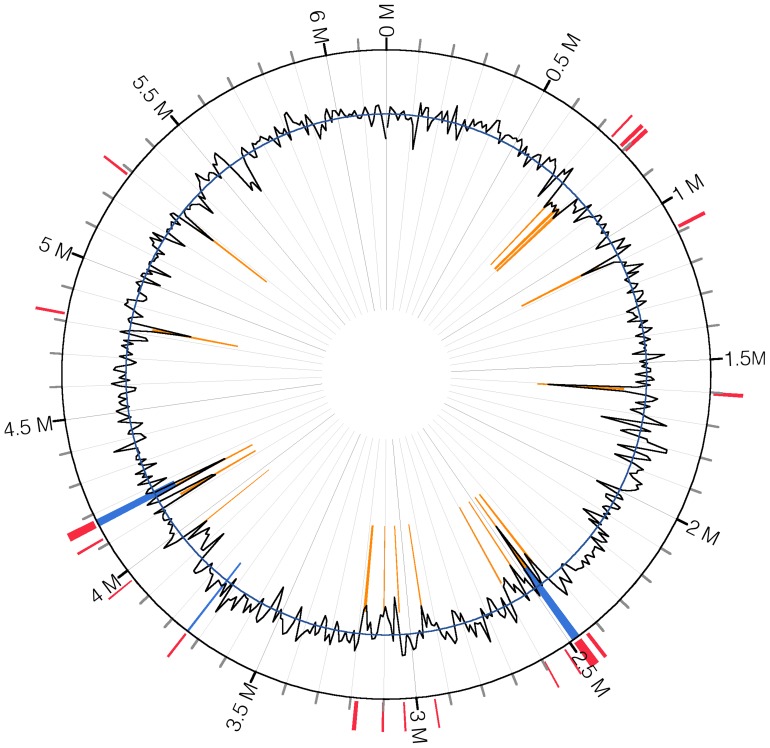
Genomic islands of *P.* sp. UW4 predicted by IslandViewer. The outer black circle shows the scale line in Mbps. Predicted genomic islands are colored based on the following methods: SIGI-HMM, orange; IslandPath-DIMOB, blue; Integrated detection, red. Black plot represents the GC content (%).

Nineteen putative GIs were identified by IslandViewer, which integrates two prediction methods IslandPath (DNA composition comparison) [25] and SIGI-HMM (codon usage) [26] (Figure 2 and Table S2). The size of the 19 putative islands ranged from 4,144 bp (GI 15) to 25,665 bp (GI 7). The largest GI 7 contains 24 genes, whereas the smallest GI 15 has 6 genes (Table S2). Eighteen GIs have a lower GC content ranging from 40.33% to 58.82% compared with the average GC content of the UW4 genome. GI 11 has a GC content of 63.92%, which is higher than the average GC content of the UW4 genome. It contains 5 genes and two of them (PputUW4_02598 and PputUW4_02599) showed high similarities (88% and 84% at the amino acid level) with those in the predicted GIs of *P. fluorescens* Pf0-1. Among the 19 GIs, six contain mobile genetic elements, such as integrase and transposase genes, suggesting that these GIs can self-mobilize [27]. The 3′ ends of tRNAs have been suggested to be hot spots for foreign DNA integration [28]. In UW4, GI 4 and GI 15 are inserted adjacent to the 3′ ends of tRNA-Leu and tRNA-Val, respectively, which support the identification of these two GIs.

The genome of *P.* sp. UW4 has 31 complete putative Insertion Sequence (IS) elements and 5 truncated remnants of IS elements (Table S3). Among the complete IS elements, sixteen belong to the IS*110* family, seven from the IS*1182* family and eight from the IS*3* family. No intact prophages were observed in the genome of UW4, nevertheless, UW4 carries 19 phage related genes (Table S4).

One hundred and eighty two tandem repeats were identified in the *P.* sp. UW4 genome (Table S5). Among the 182 repeats, 122 were found in the coding region, which may cause changes in protein expression. Sixty repeats were observed in the non-coding region, which may act as promoter components of downstream genes or transcription terminators of upstream genes [29]–[30].

In order to elucidate the protein function of the 5423 coding sequences (CDS), protein localization prediction was performed. The results indicate that UW4 consists of 2507 (46.2%) cytoplasmic proteins, 1258 (23.2%) cytoplasmic membrane proteins, 176 (3.2%) periplasmic proteins, 115 (2.1%) outer membrane proteins, and 42 (0.9%) extracellular proteins. The remaining 1325 (24.4%) CDSs have unknown localization.

### Genes Involved in Plant Growth Promotion and *P.* sp. UW4 Lifestyle

#### ACC deaminase

The ACC deaminase gene, *acdS* (PputUW4_04154), and its upstream regulatory gene, *acdR* (PputUW4_04155), were characterized previously [2], [5], [7]. The UW4 genome sequencing confirmed the presence of both genes as well as the intergenic sequences between the two genes. Interestingly, a tRNA-Arg gene was found 3,138 bp downstream of *acdR* and tRNA-Arg is one of tRNA genes that are preferentially used for insertion of GI [27]. Therefore, it is possible that the region of the genome that encodes *acdS* and *acdR* was acquired from other genera by horizontal gene transfer, which is in concert with previous findings [3]. However, this region was not identified as a GI by automatic prediction using IslandViewer.

A BLAST search of ACC deaminase genes in 20 other sequenced *Pseudomonas* genomes (Table 3) indicated that it is present in *P. brassicacearum* NFM421, *P. syringae* DC3000, *P. syringae* B728a, and *P. syringae* 1448A. Pairwise amino acid sequence identities between UW4 *acdS* and the other four genomes range from 89% to 99%, and they all contain the important active sites [48], suggesting that the putative *acdS* gene in those genomes is likely functional. Furthermore, the common *acdS* regulatory gene, *acdR*, was found immediately upstream of *acdS* in all five genomes, and the amino acid sequence identities between UW4 *acdR* and the other four genomes range from 80% to 93%. This type of *acdS* regulation scheme has been observed in many bacteria and was proposed as a main feature of the functioning of bacterial ACC deaminase [49].

**Table 3:** General Features of the *Pseudomonas* Genomes.

*Pseudomonas* sp.	Genome Size,bp	CDS no.	Pseudogenes	G+C content,%	Coding density,%	Trnano.	rRNA genes (operon no.)	Plasmidno.	Accession number	Ref.
UW4	6,183,388	5,423	20	60.05	87.2	72	22 (7)	–	CP003880	This study
aeruginosa LESB58	6,601,757	5,925	34	66.3	88.4	67	13 (4)		NC_011770	[31]
aeruginosa PA7	6,588,339	6,286	8	66.5	89.5	63	12 (4)	–	NC_009656	[32]
aeruginosa PAO1	6,264,404	5,566	5	66.6	89.3	63	13 (4)	–	NC_002516	[33]
aeruginosa UCBPP-PA14	6,537,648	5,892	none	66.3	89.4	59	13 (4)	–	NC_008463	[34]
Brassicacearum subsp. brassicacearumNFM421	6,843,248	6,097	N/A	60.8	88.2	65	16 (5)	–	NC_015379	[35]
entomophila L48	5,888,780	5,169	N/A	64.2	89.1	78	22 (7)	–	NC_008027	[36]
protegens Pf-5	7,074,893	6,144	N/A	63.3	88.8	71	16 (5)	–	NC_004129	[37]
fluorescens Pf0-1	6,438,405	5,741	9	60.6	89.8	73	19 (6)	–	NC_007492	[38]
fluorescens SBW25	6,722,539	6,009	88	60.5	88.3	66	16 (5)	–	NC_012660	[38]
mendocina NK-01	5,434,353	4,958	N/A	62.5	88.7	65	12 (4)	–	NC_015410	[39]
putida BIRD-1	5,731,541	5,124	N/A	61.7	86.9	64	22 (7)	–	CP002290	[40]
putida F1	5,959,964	5,300	49	61.9	88.7	76	19 (6)	–	NC_009512	[41]
putida GB-1	6,078,430	5,417	8	61.9	89.4	74	22 (7)	–	NC_010322	[41]
putida KT2440	6,181,863	5,420	N/A	61.6	86.7	73	22 (7)	–	NC_002947	[42]
putida S16	5,984,790	5,218	N/A	62.3	84.9	70	19 (6)	–	NC_015733	[43]
putida W619	5,774,330	5,471	26	61.4	88.9	75	22 (7)	–	NC_010501	[41]
stutzeri A1501	4,567,418	4,146	N/A	63.8	89.8	61	12 (4)	–	NC_009434	[44]
syringae pv. phaseolicola 1448A	5,928,787	5,144	N/A	58	87	64	16 (5)	2	NC_005773	[45]
syringae pv. syringae B728a	6,093,698	5,137	47	59.2	88.5	64	16 (5)	–	NC_007005	[46]
syringae pv. tomato str. DC3000	6,397,126	5,615	N/A	58.4	86.8	63	15 (5)	2	NC_004578	[47]

#### Siderophores

Siderophore production is a typical characteristic possessed by fluorescent pseudomonads. *P.* sp. UW4 fluoresces under UV light, implying the production of pyoverdine siderophore. In the UW4 genome, putative genes associated with pyoverdine synthesis are shown in Table S6 and Figure S1. The *pvdF* gene for the type I pyoverdine found in *P. aeruginosa* is absent in UW4. This gene encodes a transformylase responsible for the formation of *N^5^*-formyl-*N^5^*-hydroxyornithine from *N^5^*-hydroxyornithine [50]. However, in UW4, a gene encoding hydroxyornithine acetylase, *pvdYII*, was found. The gene product of *pvdYII* can convert *N^5^*-hydroxyornithine to *N*-hydroxy-*cyclo*-ornithine, resulting in the production of type II pyoverdine in *P. aeruginosa* [51] (Figure S2). Amino acid sequence alignment of PvdYII from UW4 and *P. aeruginosa* Pa4 (ABC55668) showed that the two proteins share 70% identities and 79% similarities, and that conservation occurs to the greatest extent at the C-terminus.

Among the other 20 sequenced *Pseudomonas* genomes, only *P. stutzeri* A1501 does not have the genes for siderophore biosynthesis, and it also has the smallest genome compared to the other 20 species, suggesting loss of functions in A1501 [44]. Compared with UW4, 11 genomes contain a gene encoding PvdYII including *P. putida* KT2440 (locus_tag: PP_4245), *P. putida* BIRD-1 (PPUBIRD1_1611), *P. putida* F1 (Pput_1682), *P. putida* GB-1 (PputGB1_3811), *P. putida* W619 (PputW619_3564), *P. putida* S16 (PPS_3636), *P. brassicacearum* NFM421 (PSEBR_a1665), *P. entomophila* L48 (PSEEN_1813), *P. fluorescens* Pf0-1 (Pfl01_3942), *P. mendocina* NK-01 (MDS_1799), *P. aeruginosa* PA7 (PSPA7_2826), indicating these species likely produce type II pyoverdine since this gene was only observed in the strains of *P. aeruginosa* that make type II pyoverdine [51]–[52]. However, the precise structure of the siderophore needs to be confirmed experimentally.

In *P. protegens* Pf-5, genes responsible for pyoverdine as well as pyochelin were identified. The genes required for pyoverdine synthesis are located in three clusters whereas genes necessary for pyochelin synthesis are present in a single cluster [37]. In *P. entomophila* L48, two gene clusters of pyoverdine synthesis are present, which exhibits similar organization compared with that found in other fluorescent pseudomonads. In addition, one gene cluster related to acinetobactin was observed on the chromosome, and it contains a salicylamide moiety [36]. *P. syringae* DC3000 produces two types of siderophores, pyoverdine and yersiniabactin, and in both cases the required genes are present in a single cluster [47]. *P. syringae* B728a also secretes two types of siderophores. The first type is pyoverdine, and as in DC3000, the determinants are located in one gene cluster. The second type is achromobactin, which is a citrate siderophore produced by *Pectobacterium chrysanthemi* and *Escherichia carotovora* pv. *atroceptica* [46]. The ability of bacteria to produce multiple siderophores surely benefits these organisms, as they may function in different environments, making them more competitive against other organisms in the same niche.

#### IAA

Although many bacteria are able to synthesize IAA, the amounts produced vary significantly between strains. Depending on the concentration, bacterially produced IAA can either stimulate or inhibit plant growth. It was demonstrated previously that *P.* sp. UW4 actively produces the phytohormone IAA [53]. Here, two potential IAA biosynthesis pathways, the indole-3-acetamide (IAM) and indole-3-acetonitrile (IAN) pathways, were identified in the genome of UW4, and 6 genes might be involved (Figure S3). However, the indole-3-pyruvate pathway, which was identified in another PGPB, *Pseudomonas putida* GR12-2 [54], is absent in UW4. In the IAM pathway, tryptophan is converted to IAM by tryptophan 2-monooxygenase (PputUW4_04962) and then to IAA by amidase (PputUW4_03350). In the IAN pathway, tryptophan is converted to indole-3-acetaldoxime and then to IAN by indoleacetaldoxime dehydratase (PputUW4_03348). Next, IAA can be produced directly through IAN by nitrilase (PputUW4_02461). Alternatively, IAN can be first converted to IAM by nitrile hydratase (PputUW4_03351 and PputUW4_03352), and then IAM is converted to IAA by amidase (PputUW4_03350). Future work will involve an experimental confirmation of the putative functions of the above-mentioned genes in IAA biosynthesis.

A search of the sequenced *Pseudomonas* genomes for UW4-like IAA pathway associated genes revealed the presence of 6 orthologous genes in *P. fluorescens* SBW25 and *P. putida* F1, suggesting similar IAA synthesis pathways compared to UW4. *P. putida* BIRD-1 has 5 homologs that complete the IAM and IAN pathways, but it lacks the gene encoding nitrilase (PputUW4_02461).

Many studies have shown that numerous bacterial strains possess multiple IAA synthesis pathways. Besides the above-mentioned strains, it has been observed that putative tryptamine and IAM pathways are present in *P. putida* W619, GB-1, and F1 [41]. Therefore, to study the role of each gene in IAA biosynthesis of a particular bacterium it is necessary to construct a large number of mutants, single or multiple, and test the function of each one.

#### Trehalose

Trehalose is a non-reducing disaccharide of glucose whose two glucose moieties are linked by an α,α-1,1-glycosidic bond. It functions as an osmoprotectant in the stabilization of biological structures including dehydrated enzymes, proteins and lipids under environmental stresses such as drought, high salinity and low temperature in a wide range of organisms, i.e. bacteria, archaea, fungi, invertebrates, insects and plants. In transgenic rice, trehalose improves the plant’s abiotic stress tolerance [55]. In another study, when maize plants were inoculated with a strain of *Azospirillum brasilense* transformed to overexpress trehalose, 85% of the plants survived drought stress, whereas only 55% of the plants inoculated with the non-transformed strain survived. Furthermore, a 73% increase in the biomass of maize plants was obtained when the plants were inoculated with the transformed strain [56]. In bacteria, five trehalose biosynthetic pathways are known including OtsA/OtsB, TreS, TreY/TreZ, TreP, and TreT [57]. In the genome of UW4, two trehalose synthesis pathways, TreS and TreY-TreZ pathways, were identified. The TreS pathway involves the conversion of maltose to trehalose by trehalose synthase (TreS) (PputUW4_02800). In the TreY-TreZ pathway, maltodextrin is first converted to maltooligosyltrehalose by maltooligosyltrehalose synthase (TreY) (PputUW4_02792), and then to trehalose by maltooligosyltrehalose trehalohydrolase (TreZ) (PputUW4_02790). When searching the orthologs in the other *Pseudomonas* genomes, all 20 species contain the genes involved in those two trehalose synthesis pathways, and they are organized in a similar way, indicating the ubiquity and importance of this sugar. In addition, *P. stutzeri* A1501 has a third trehalose synthesis pathway, OtsA/OtsB, which is the most widespread pathway present in both eukaryotes and prokaryotes, and this may further contribute to its survival under different environmental stresses.

#### Acetoin

Acetoin is volatile compound released from certain PGPB, which can promote plant growth by stimulating root formation [58]. In the genome of UW4, genes involved in acetoin production were identified, including acetolactate synthase (PputUW4_04612 and PputUW4_04613) and zinc-containing alcohol dehydrogenase (PputUW4_03046). However, acetoin reductase that is responsible for the conversion of acetoin to 2,3-butanediol is absent from the UW4 genome.

When the genomes of the other *Pseudomonas* were examined for acetoin synthesis, the same pathway was observed in all 20 species, although the enzyme that catalyzes the last step could not be determined definitely due to ambiguous annotations.

#### Antimicrobial compounds and antibiotics resistance

It was reported that 4-hydroxybenzoate has antimicrobial activity and its biosynthesis pathway was found in the genome of several PGPB, such as *Pseudomonas protegens* Pf-5 [37], *Enterobacter* sp. 638 [59] and *Mesorhizobium amorphae* [60]. In bacteria, 4-hydroxybenzoate is formed from chorismate directly by chorismate lyase encoded by *ubiC*. A search for the gene *ubiC* in *Pseudomonas* genomes determined that it was present in all 21 species including UW4 (PputUW4_05351), suggesting 4-hydroxybenzoate synthesis is a common pathway in *Pseudomonas* spp.

Antibiotic susceptibility testing of UW4 has shown that it is resistant to ampicillin (128 µg/ml), erythromycin (64 µg/ml), and novobiocin (256 µg/ml). Two genes that encode β-lactamase were found in the UW4 genome (PputUW4_01223 and PputUW4_01636), which may confer the ampicillin resistance of the strain. One gene that encodes macrolide glycosyltransferase (PputUW4_03146) was identified; the product of this gene can glycosylate and inactivate macrolide antibiotics such as erythromycin [61]. Novobiocin is produced by *Streptomyces* and this antibiotic’s target is DNA gyrase subunit B [62]–[63]. There are two mechanisms used by bacteria to inhibit novobiocin activity. One strategy is through the mutation of the gyrase B (*gyrB*) subunit gene [64]. For example, *Streptomyces sphaeroides* has two *gyrB* genes and one is novobiocin sensitive, which is constitutively produced, and the other one is novobiocin resistant, which is induced by the drug [65]. The second strategy of novobiocin resistance is through the use of multidrug efflux pumps [66]. *P.* sp. UW4 has a single *gyrB* gene (PputUW4_00004) in its genome. The product of this gene has not been characterized to determine if it is novobiocin sensitive or resistant. On the other hand, multiple multidrug efflux systems have been identified in the UW4 genome based on sequence similarity search, which may play an important role in novobiocin resistance (Table S7).

### Polyhydroxyalkanoates

Polyhydroxyalkanoates (PHAs) are a group of metabolic energy and carbon storage compounds that are deposited as intracellular water-insoluble granules in many living organisms during imbalanced growth conditions [67]. PHAs extracted from bacteria can be used as alternative starting materials to petrochemical in the synthesis of plastics because they are biodegradable and environmentally friendly [68]. Furthermore, bacteria can accumulate PHAs to levels as high as 90% (w/w) of the dry cell mass, making them potential candidates for the large-scale production of PHAs [69]. Recently, it has been reported that PHA production played an important role in cold adaptation of an Antarctic bacterium *Pseudomonas* sp. 14-3, likely by alleviating the oxidative stress induced by cold environments [70]. Thus, the PHA synthase-minus mutant of *Pseudomonas* sp. 14-3 could not grow at 10°C and was more susceptible to freezing than the wild-type strain. In addition, cold shock treatment caused rapid degradation of PHA in the wild-type strain [70].

The genes involved in PHA synthesis are found in many *Pseudomonas* sp. such as *P. putida* KT2440, *P. putida* GPo1, *P. aeruginosa* PAO1, *P. fluorescens* Pf0-1, *P. protegens* Pf-5, *P. syringae* pv *phaseolicola*, and *P. syringae* DC3000 [71]. The gene cluster typically contains six genes including *phaC1, phaZ, phaC2, phaD, phaF*, and *phaI*. The order of the six genes is highly conserved (*phaC1ZC2DFI*) in the above-mentioned strains and was also observed in UW4 (PputUW4_00333–00328). The gene *phaC* encodes the key enzyme, PHA synthase or PHA polymerase, for the biosynthesis of PHA. The PhaC1 and PhaC2 belong to the class II PHA synthases that preferentially use 3-hydroxyalkanoates consisting of 6–14 carbons as substrates, and the class II PHA synthases are primarily found in *Pseudomonas* spp. The *phaZ* gene encodes a depolymerase that is responsible for PHA degradation. The gene product of *phaD* is a transcriptional regulator that positively regulates the expression of the downstream genes, *phaI* and *phaF*, which code for phasins [71]. When this *pha* gene cluster was searched against the other *Pseudomonas* genomes, orthologs were found to be absent in *P. syringae* pv. *syringae* B728a. In addition, the genome of *P. stutzeri* A1501 contains a gene cluster different from *phaC1ZC2DFI*, designated *phaCABR* that is responsible for poly-hydroxybutyrate (PHB) synthesis [44]. In the genome of UW4, a second *phaC1* gene was identified (PputUW4_02300). Compared with the *phaC1* in the *pha* gene cluster, the second *phaC1* showed 69% identities and 83% similarities. It is likely that the redundant *phaC1* gene also contributes to the production of PHA in UW4, however this has to be confirmed experimentally.

#### Degradation of aromatic compounds

In polluted environments, *P. putida* strains are often isolated as predominant microorganisms and are therefore commonly used in bioremediation. Aromatic compounds are among the most abundant and recalcitrant pollutants in the soil and their degradation by bacteria usually involves ring-cleavage in the presence of O_2_ by oxygenase [72]. For example, the toluene degradation pathway in *P. putida* F1 is composed of the toluene dioxygenase operon *todABC1C2DE* [73]. However, this toluene degradation pathway is absent in all of the other 20 *Pseudomonas* sp., as well as UW4. In the genome of *P. putida* W619, the genes involved in 3-HPP were identified previously, and the complete pathway includes the enzymes encoded within the *mhpRABCDFET* operon (Wu et al. 2011). Nevertheless, this pathway seems unique in this strain because in the other 20 *Pseudomonas* genomes, it is either absent or incomplete, such as in *P. putida* F1 [41] and UW4 (five putative enzyme-encoding genes, *mhpACDFE*, were found) (PputUW4_02144, PputUW4_01659–01662). In the genome of UW4, a complete degradation pathway of benzoate via the catechol route of the β-ketoadipate pathway was identified. In addition, the protocatechuate branch of the β-ketoadipate pathway is also present. Protocatechuate is one of the key intermediates during the degradation of various aromatic compounds, including 4-hydroxybenzoate and quinate [74]. Since this pathway is considered to be one of the central pathways for the catabolism of aromatic compounds in *Pseudomonas* spp., its presence is ubiquitous in the completely sequenced *Pseudomonas* genomes.

#### Heavy metal resistance

Based on the genome sequence of *P.* sp. UW4, various heavy metal resistance determinants were identified (Table S8). It has been shown that UW4 can grow in rich medium containing 2 mM nickel at a growth rate of 0.24 generations/hour [20]. As expected, putative nickel transporters were found in the UW genome. The genes encoding the transporters showed similarities to the Nik system (*nikABCDER*) that was originally identified in *E. coli*. In the genome of UW4, the locus of the Nik system contains three copies of NikA (PputUW4_00743, 00745, 00746) and a single copy of NikB (PputUW4_00742), NikC (PputUW4_00741), NikD (PputUW4_00740), and NikE (PputUW4_00739). However, based on a sequence similarity search, NikR, a nickel-responsive regulator, is not encoded in the UW4 genome.

Sixteen genes that might be involved in the copper resistance of UW4 were identified (Table S8). These 16 genes are located at six regions on the chromosome, including three copper resistance systems in UW4, two individual sets of two-component transcriptional regulators, and one gene that might be involved in the bacterium’s survival in the presence of high bioavailable Cu(II). The first region contains three genes (PputUW4_00578, 00579, 00581), resembling the CueAR-CopP in *P. putida* PNL-MK25, which has been experimentally confirmed to play an important role in copper homeostasis [75]. The second region related to copper resistance contains four genes (PputUW4_03484–03487). The homologs for the four genes are designated *copABCD* [76]. CopA is a multi-copper oxidase family protein [77]. CopB is a protein involved in copper binding. The gene encoding CopC is similar to periplasmic proteins involved in copper resistance, and the last gene in the operon is *copD,* which encodes a copper transport protein. The third copper resistance locus consists of four genes, *cinQARS* (PputUW4_03498–03501). The gene *cinQ* encodes a putative 7-cyano-7-deazaguanine (pre-Q_0_) reductase, and *cinA* encodes a putative copper-containing azurin-like protein. The gene products of the *cinRS* operon are a two-component heavy metal response transcriptional regulator (CinR) and a heavy metal sensor histidine kinase (CinS). Sequence analysis of the two sets of two-component transcriptional regulators PputUW4_02046–02047 and PputUW4_04493–04494 showed similarities compared with CopRS in *P. putida* KT2440. The *copR* gene encodes a two-component heavy metal response transcriptional regulator and *copS* encodes a heavy metal sensor signal transduction histidine kinase. Lastly, a protein that might be involved in bacterial survival in the presence of high bioavailable Cu(II) was identified in the genome of UW4 (PputUW4_02449). A sequence similarity search showed that it has high similarities compared with CopG1 and CopG2 in KT2440. Both *copG1* and *copG2* are located within copper resistance operons in KT2440. However, this is not observed in the UW4 genome.

Besides nickel and copper, *P.* sp. UW4 may possess resistance to other heavy metals, such as cadmium, zinc, cobalt, molybdenum, chromate, and arsenate. Two genes, *cadA1* (PputUW4_05166) and *cadR* (PputUW4_05167), involved in cadmium resistance were identified. The gene *cadA* is known to encode a cadmium-transporting ATPase, and CadR is a MerR family response regulator responsible for cadmium resistance. In the genome of UW4, another *cadA* gene (PputUW4_05407) was identified based on a sequence similarity search. However, when comparing the amino acid sequence of the PputUW4_05407 to CadA from *P. putida* 06909, the identities and similarities are only 36% and 52%, respectively. Furthermore, PputUW4_05407 lacks the HMA domain at N terminus. Therefore, the function of CadA in UW4 needs to be confirmed experimentally.

Zinc is an essential trace element that acts as a cofactor for many enzymes. However, high concentrations of zinc are toxic to the cell. Bacteria employ different strategies to control zinc levels, including storage by metallothionein and export from the cell by ABC transporter systems [78]. In the genome of UW4, a putative metallothionein was identified (PputUW4_01616). In addition, a common zinc transporter system is also present in UW4. The system consists of three genes *znuABC* (PputUW4_00067, 00064, 00065) and one transcriptional repressor *zur* (PputUW4_00066). The gene products of *znuABC* are a periplasmic binding protein, a membrane permease, and an ATPase, respectively. The gene *zur* is located between *znuA* and *znuC*, and is transcribed in the same orientation as *znuBC*, but in the opposite direction from *znuA*.

The molybdate transport system in *P.* sp. UW4 is comprised of three genes, *modABC* (PputUW4_02399, 02398, 02397). ModA is a periplasmic binding protein; ModB is an integral membrane protein; and ModC is an ATPase. In *E. coli*, the *modABC* expression is tightly controlled by a repressor protein, ModE, and the gene is located upstream of *modABC* operon [79]. In the genome of UW4, a homolog of ModE is not present upstream of the molybdate transport system. However, a ModE family transcriptional regulator (PputUW4_04985) is found elsewhere on the chromosome.

One cobalt transporter locus comprising two genes, *cbtA* (PputUW4_02359) and *cbtB* (PputUW4_02360) was identified in the genome of UW4. This transport system has been found in various bacteria and it is related to vitamin B_12_ biosynthesis. Homologs of CbtA usually have five transmembrane segments, and the gene is always co-localized with *cbtB*, which encodes one transmembrane segment and a histidine-rich C terminus likely to be a metal-binding site [80].

Arsenic ions are very toxic to most microbes and are common environmental pollutants. Arsenic resistance determinants were found in three regions on the chromosome of UW4, including an operon *arsRBCH* (PputUW4_02251–02248), and two individual *arsC* genes (PputUW4_01082 and PputUW4_04117). The gene product of *arsC* is an arsenate reductase that catalyzes the reduction of arsenate to arsenite. ArsB is an arsenite efflux transporter, which can extrude arsenite out of the cell. ArsR functions as an arsenical resistance operon repressor that responds to arsenate [81]. ArsH is a NADPH-dependent FMN reductase and its role in arsenic resistance is not clear [82]. The other two individual ArsC proteins showed far less similarity compared with the ArsC in the operon, implying that they belong to different families of arsenate reductase.

The mechanism used by various bacteria to extrude toxic chromate is through a chromate transporter, ChrA [83]–[87]. A gene encoding ChrA was identified in the UW4 genome (PputUW4_03067), and the protein sequence of ChrA in UW4 showed 75.8% identities when compared with the gene from strain KT2440. In another study, a small protein, OscA, was found to be responsible for chromate resistance in *Pseudomonas corrugata* 28 [88]. In the genome of UW4, an *oscA* homolog (PputUW4_00153) was found upstream of a sulfate-binding protein gene (*cysP*), which has been demonstrated to form a transcriptional unit with *oscA* [88]. The genetic organization of the *oscA* region is exactly the same as this region in *P. corrugata* 28, indicating that the *oscA* gene from UW4 may also play an important role in chromate resistance.

#### Central metabolic pathways

**Figure 3:**
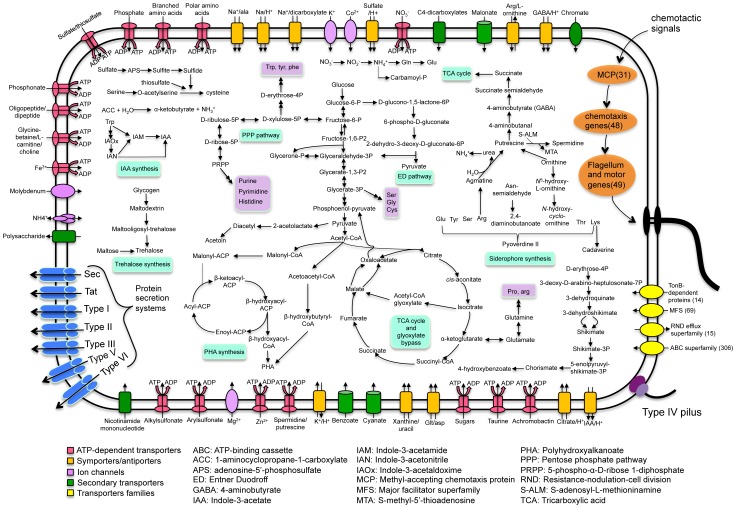
Schematic overview of metabolic pathways and transport systems in *P.* sp. UW4. Individual pathways are denoted by single-headed arrows, while reversible pathways are denoted by double-headed arrows.

A schematic summary of the metabolic strategies in *P.* sp. UW4 is shown in Figure 3. The genome of *P.* sp. UW4 contains a complete central carbon metabolism pathway including glycolysis/gluconeogenesis, a tricarboxylic acid (TCA) cycle with glyoxylate bypass, and a pentose phosphate pathway (PPP).

Metabolism of sulfur in UW4 involves assimilation of inorganic sulfate and mineralization of organic sulfonates. Inorganic sulfate or thiosulfate is transported into the cell by an ABC-type transporter including a periplasmic binding protein, Sbp (PputUW4_03826) for sulfate or CysP (PputUW4_00154) for thiosulfate, permease CysT and CysW, and an ATPase CysA. Sulfate and thiosulfate use the same permease components and ATPase for transport. Once in the cell, sulfate is activated to adenosine-5′-phosphosulfate (APS) by sulfate adenylyltransferase, CysDN (PputUW4_00795, 00796), and then to sulfite by phosphoadenosine phosphosulfate reductase, CysH (PputUW4_03665). Sulfite is further reduced to sulfide by sulfite reductase, CysI (PputUW4_02350). This sulfide then joins *O*-acetylserine catalyzed by cysteine synthase, CysK (PputUW4_04003) to form cysteine. In the case of thiosulfate, a gene encoding *O*-acetylserine sulfhydrylase, CysM (PputUW4_04107), catalyzes the reaction between thiosulfate and *O*-acetylserine to generate *S*-sulfocysteine, which is then converted to cysteine [89]–[90]. In the genome of UW4, six SulP family sulfate transporters were identified (PputUW4_00023, 00047, 00617, 02916, 03092, 04194). Although the role of these transporters in sulfate assimilation in bacteria is not clear, the homologs in several eukaryotes have been characterized and shown to be active components of sulfate transport, some of which function as sulfate:H^+^ symporters [91]. Organosulfur compounds are widely present in nature. For example, in aerobic soils organic sulfur can make up greater than 95% of the total sulfur in the forms of peptides/amino acids, sulfonates (C-SO_3_H), sulfamates (C-NH-SO_3_H), and sulfate esters (C-O-SO_3_H) [89]. Desulfonation of alkanesulfonates by UW4 is potentially catalyzed by alkanesulfonate monooxygenase, SsuD (PputUW4_05211), and an NADPH-dependent FMN reductase, SsuE (PputUW4_05213). The two genes are located within an operon, *ssuEADCBF* (PputUW4_05208–05213), which also includes sulfonate transporter genes, *ssuABC*, and a molybdenum-pterin binding protein gene, *ssuF*. Similar to other *P. putida* strains, a gene encoding the thiol-specific antioxidant, LsfA, was found upstream of *ssuE*. It has been demonstrated that expression of *lsfA* increased dramatically under sulfate starvation [92]. Taurine is a naturally occurring aliphatic sulfonate. In the genome of UW4, two operons that each contains four genes encoding an ABC-transporter (*tauABC*) (PputUW4_00119–00121 and PputUW4_00198–00200) and a taurine dioxygenase (*tauD*) (PputUW4_00118 and PputUW4_00197) were identified. In addition, a third set of genes *tauA* (PputUW4_05218) and *tauD* (PputUW4_00894) are present in the genome. However, neither of them is associated with other *tau* genes. Like sulfonates, sulfate esters are components commonly present in soil. A sulfatase gene cluster that might be involved in desulfurization of aryl and alkylsulfate esters of UW4 was identified. The cluster contains seven genes, *atsACBR-sftR-atsK-sftP* (PputUW4_00164–00170), which encode arylsulfatase, sulfate ester transporter ATP-binding component, aliphatic sulfonates ABC transporter permease, periplasmic aliphatic sulfonates-binding protein, LysR family transcriptional regulator, alkylsulfatase, and TonB-dependent receptor, correspondingly. It has been reported that in many gram-negative bacteria a LysR-type transcriptional regulator, CysB, mediated global sulfur regulation. Under the sulfur limitation conditions, CysB activates the transcription of cysteine synthesis genes in the presence of *N*-acetylserine or *O*-acetylserine, whereas sulfide and thiosulfate function as corepressors by inhibiting the binding of CysB to the promoters of the cysteine synthesis genes [93]–[95]. In UW4, a gene encoding CysB was identified (PputUW4_01421) and it contains a typical helix-turn-helix motif at the N terminus for binding to the target DNA.

*P.* sp. UW4 is unable to fix nitrogen and it also lacks the genes for denitrification. However, it contains the genes for assimilatory nitrate reduction. Two types of nitrate transporters are present on the chromosome of UW4 including an ABC-type nitrate transporter system and a NarK family transporter NasA. The locus of the ABC transporter system contains three genes that encode a nitrate transporter periplasmic protein (PputUW4_02319), a nitrate transporter permease (PputUW4_02320), and a nitrate transporter ATP-binding protein (PputUW4_02321). NasA is located within a cluster of eight genes, *nasST-nasA-ppkB-nasDEC-cobA* (PputUW4_03638–03645), which is potentially involved in nitrate/nitrite assimilation. The gene *nasS* encodes a periplasmic nitrate-binding protein and *nasT* encodes a response regulator that acts as an inducer of the *nas* operon in response to the presence of nitrate/nitrite [96]–[97]. It has been shown that NasA is a nitrate transporter and a *nasA* mutant was unable to grow on nitrate but capable of growing on nitrite [98]. The genes *nasDEC-cobA* are located within an operon and they encode assimilatory nitrite reductase (NasDE), assimilatory nitrate reductase (NasC), and uroporphyrin III methyltransferase (CobA), respectively. Uroporphyrin III methyltransferase is an enzyme responsible for siroheme synthesis and the gene was induced strongly by nitrate [99]. Furthermore, a siroheme synthetase homolog gene mutant of *Rhizobium etli* was unable to grow on nitrate as the sole nitrogen source [100]. The gene *ppkB* (PputUW4_03641), which is located immediately downstream of *nasA*, encodes a serine/threonine protein kinase. It has been demonstrated that a protein kinase carried out phosphorylation of the nitrate transporter and played an important role in nitrate deprivation response in *A. thaliana* and *Hansenula polymorpha* [101]–[102].

Many soil bacteria are capable of solubilizing poorly soluble mineral phosphates by synthesizing organic acids and acid phosphatases. In the genome of UW4, the genes responsible for gluconic acid synthesis were found. The production of gluconic acid is catalyzed by glucose dehydrogenase (PputUW4_00989) and its cofactor PQQ. The PQQ biosynthetic genes of UW4 are clustered in two separate loci on the chromosome: the *pqqABCDEFH* (PputUW4_04964–04970) and the *pqqBCDE* (PputUW4_02938–02941). In addition, five putative acid phosphatase-encoding genes were identified including two phosphatidic acid phosphatase (PAP2) protein genes (PputUW4_00631, 04385), two SurE superfamily protein genes (PputUW4_01116, 01671), and one non-specific acid phosphatase gene (PputUW4_02824). However, no phytase gene is present in UW4. Inorganic phosphate uptake in UW4 may be facilitated by two high-affinity phosphate transport systems: PstBACS (PputUW4_05361–05364) and PhnDCE1E2 (PputUW4_03163–03166), and one low-affinity phosphate transport system, PitA (PputUW4_01197). The high-affinity phosphate uptake system is composed of multi-subunit ABC transporters and is induced by phosphate-starvation, whereas the low-affinity system consists of a single membrane protein and is constitutively expressed [103].

### Secretion Systems

*P.* sp. UW4 has seven pontential protein secretion systems including Sec, Tat, Type I, II, III, V and VI (Table S9).

The Sec (general secretory pathway) and Tat (twin arginine translocation) systems are the two ubiquitous systems for export across the cytoplasmic membrane. UW4 has one of each such system. MscL is a large conductance mechano-sensitive channel protein and is able to export small proteins in response to osmotic pressure changes within the cell [104].

Type I secretion system (T1SS) consists of an outer membrane protein, an ABC transporter, and a membrane fusion protein. Three complete T1SS and their putative substrates were identified in UW4 (PputUW4_00114, 00115–00117, 01719–01722, 03950–03953). In addition, one partial T1SS containing only an ABC transporter and a membrane fusion protein was found (PputUW4_02631–02633). The putative substrate, mannuronan C-5-epimerase, is located downstream of the membrane fusion protein and is transcribed in an opposite direction. Since this system lacks the outer membrane protein, the transport mechanism of this large extracellular protein (1871 aa) is not clear.

Genes involved in the type II secretion system (T2SS) of UW4 are located mainly within one cluster consisting of two separate operons (PputUW4_03282–03290 and PputUW4_03297–03298). The first operon contains nine genes but only five can be identified as T2SS protein genes based on sequence similarities. The other four genes encode three hypothetical proteins and a fimbrial assembly protein.

A potential type III secretion system (T3SS) was observed in UW4 which consists of 26 genes, with 25 genes located in one cluster (PputUW4_03613–03637), and one gene encoding a HopJ type III effector located elsewhere (PputUW4_00807). Sequence analyses showed that the gene cluster (PputUW4_03613–03637) is highly similar to the SPI-1 found in a PGPB, *P. fluorescens* F113 [105]. However, the function of the T3SS in strain F113 has not been demonstrated experimentally. T3SS was found in other PGPB as well. For example, *P. fluorescens* SBW25 has a 20-kb cluster containing 22 CDSs of T3SS-related genes [106]. This system resembles the T3SS of *P. syringae* at the level of amino acid sequence and with respect to genomic organization. Although the wild-type SBW25 is a PGPB and does not induce a hypersensitive response (HR) in host plants, a modified strain that over-expressed the sigma factor RspL specific to T3SS did elicit HR in *A. thaliana* and *Nicotiana clevelandii* [106]. Four other *P. fluorescens* strains also contain a T3SS including WH6, KD, Q8r1-96, and BBc6R8 [107]–[111]. WH6 seems to have a complete and functional T3SS (PFWH6_0718–0737) consisting of 20 genes, and it is highly homologous to the T3SS region of *P. syringae* [107]. The T3SS of the biocontrol strain KD is also thought to originate from *P. syringae*. It has been demonstrated that this T3SS is functional in KD, and the T3SS mutant of KD had low biocontrol activity against *Pythium ultimum* on cucumber while maintaining its root-colonization ability [109]. Similar to SBW25 and KD, the strain Q8r1-96 has a functional T3SS with a *P. syringae* origin. However, the genomic organization of the gene cluster is divergent from SBW25 and KD [110]. Strain BBc6R8 is a Mycorrhiza Helper Bacterium (MHB), which promotes ectomycorrhizal symbiosis between Douglas fir roots and *Laccaria bicolor* [111]. It was found that the T3SS mutants were incapable of promoting mycorrhization. Although the T3SS has been most studied in terms of bacterial pathogenicity, there is increasing evidence showing that it is actually beneficial for plant health and nutrition [106]–[114]. Therefore, the prescence of a T3SS in UW4 is not supprising and it will be interesting to investigate its functionality.

The type V secretion system (T5SS) of Gram-negative bacteria contains two steps: inner membrane transport via Sec pathway and outer membrane transport by a β-barrel protein. Currently, two subtypes of T5SS have been identified including the autotransporters (ATs) and the two-partner secretion system (TPS). In UW4, three putative ATs were found. One of them, *estA* (PputUW4_04920), possesses esterase activity and was shown to play an important role in twitching, swarming, and swimming motilities of *P. aeruginosa* [115]. The other two putative ATs in UW4 encode an outer membrane autotransporter (PputUW4_02797) and an extracellular serine protease (PputUW4_00217), respectively. However, none of these have been characterized experimentally. The TPS system consists of two proteins. One protein, TpsA, has a secretion motif and a catalytic domain. The other protein, TpsB, contains the β domain involved in recruitment of the TpsA protein. Several TPS systems have been identified in *Pseudomonas* spp. such as *P. aeruginosa* PAO1, *P. fluorescens* Pf0-1 and *P. putida* KT2440 [116]–[117]. However, none of those systems is present in UW4.

The type VI secretion system (T6SS) was first described in *Vibrio cholerae* [118]. Since then, the T6SS has been found in the genome of hundreds of bacteria, where it reportedly functions as a regulator of bacterial interactions and competition [119]. UW4 contains one gene cluster that is associated with T6SS. The cluster is composed of twenty genes (PputUW4_03071–03090) including the core components to form the minimal apparatus. Haemolysin coregulated protein (Hcp) forms hexamers and eventually assembles as nanotubes, which are responsible for transportation of other T6SS effector proteins. Another protein, valine-glycine repeat protein (VgrG), forms a trimer and serves as a puncturing device towards the targeted cells. Structures of Hcp and VgrG indicated that they are related to the needle tail and syringe components of bacteriophage T4. TssB (Type Six Secretion B) and TssC form structures similar to the bacteriophage needle sheath, and TssE resembles the needle hub. TssM, TssL, and TssJ are three proteins anchored to the bacterial cell envelope. TssJ, an outer membrane lipoprotein, interacts with the inner membrane protein TssM, which links the inner and outer membrane, and forms a stable complex with protein TssL [119]–[121].

Efficient plant growth stimulation requires effective root colonization that often relies on the bacterial cell surface structures, such as pili. Type IV pili are 5–7 nm fibers and the function is controlled by numerous genes. A total of twenty-four genes that are involved in type IV pili biosynthesis were identified on the genome of UW4 (Table S10). These genes are arranged mainly within four clusters, *pilMNOPQ*, *pilACD*, *pilEXWV-fimT*, *pilL/chpA-pilJIHG*, where the last cluster contains the genes involved in pili biosynthesis regulation.

### Genome Comparisons and Phylogeny

**Figure 4:**
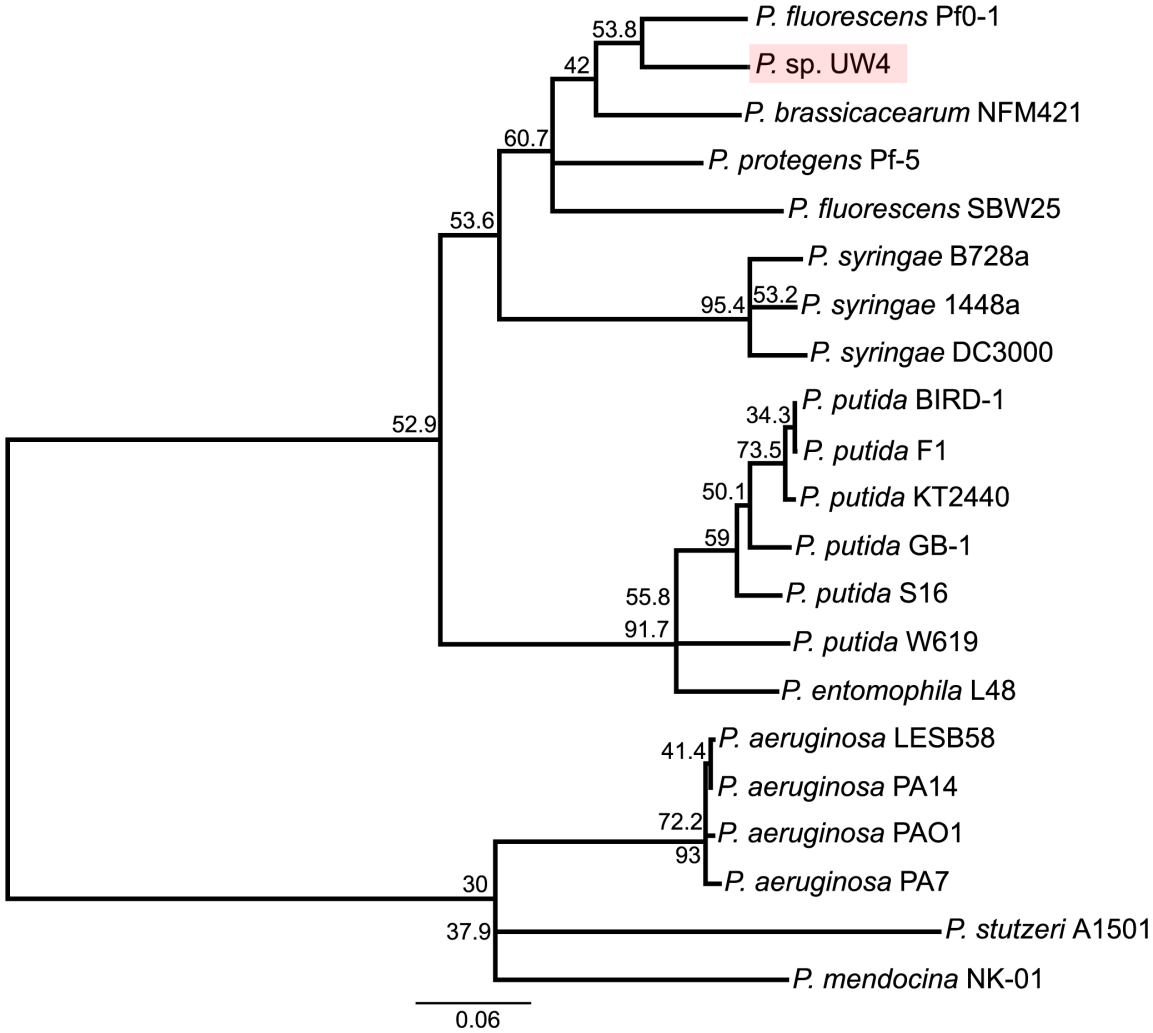
Phylogenetic tree of 21 different *Pseudomonas* species, base on 1,679 conserved genes. Numbers on nodes represent percentages of individual trees containing that relationship. The scale bar corresponds to the number of substitutions per site.

A total of 1679 orthologous genes were identified between *P.* sp. UW4 and 20 other completely sequenced *Pseudomonas* genomes. Phylogenetic analysis of the 1679 conserved genes indicated that *P.* sp. UW4 has a closer relationship with *P. fluorescens* than with *P. putida* (Figure 4). The putative orthologous relations between UW4 and 20 completely sequenced *Pseudomonas* genomes are shown in Table S11, with *P. fluorescens* Pf0-1 and *P. protegens* Pf-5 being the top two. In addition, 71 CDSs were found in other *Pseudomonas* strains, whose genome sequences have not been determined (Table S12). In UW4 genome, 271 CDSs were considered as unique based on two criteria: 1. No hits to any CDS present in NCBI nr protein sequences database with a cutoff E-value of 1 E^-20^; 2. Identities are less than 30% and/or query/subject coverage is less than 80% (Table S13). Among the 271 CDSs, 239 have been annotated as hypothetical proteins. When comparing UW4 CDSs with those in nr database, 199 showed similarities with protein sequences in other genera only, indicating these genes probably originated from a genus outside of *Pseudomonas* (Table S14).

**Figure 5:**
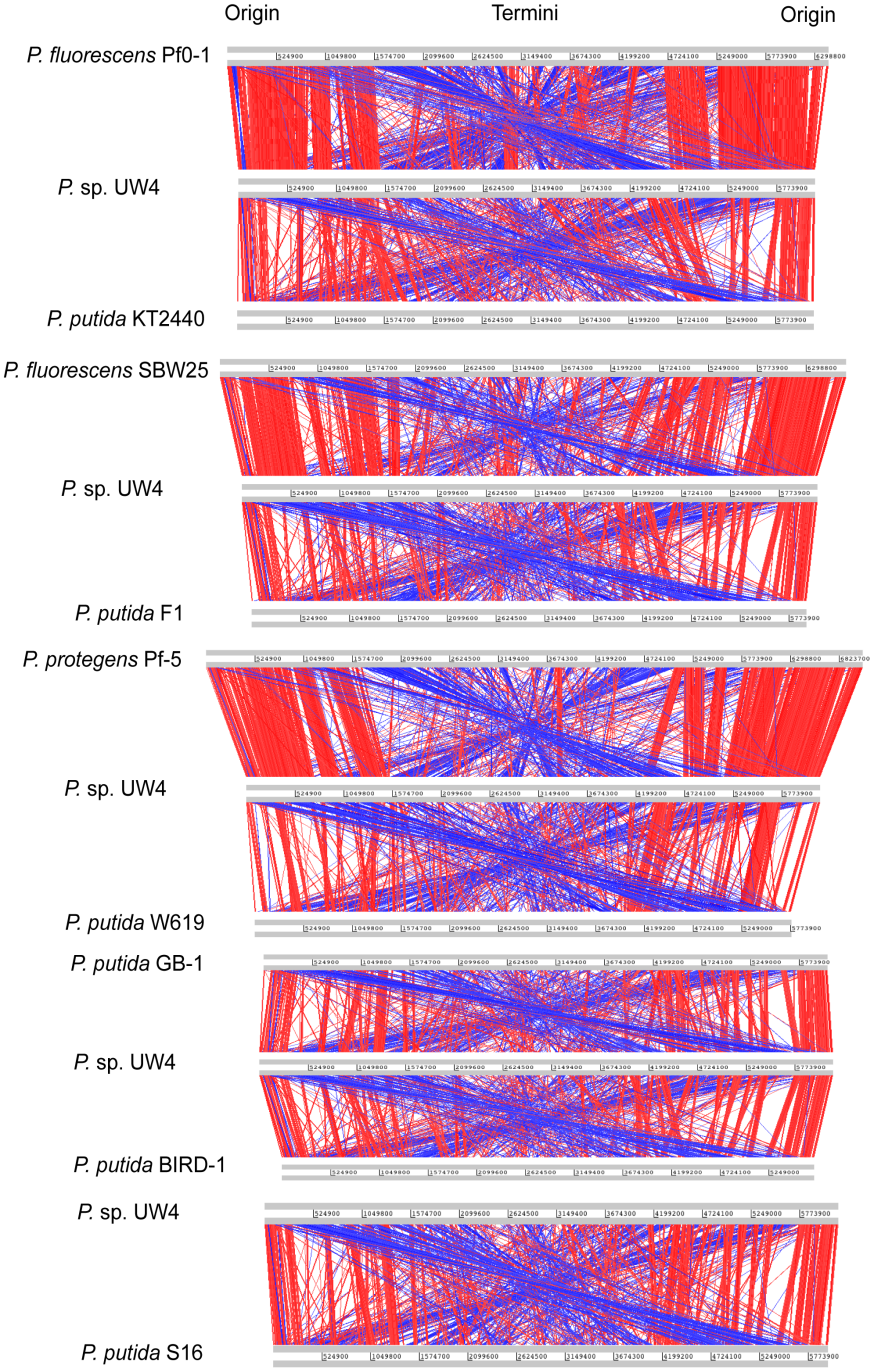
Comparative synteny line plots of the complete six-frame translations of the whole genome sequences of *P.* sp. UW4 with other *P. fluorescens*. *P. protegens*, and *P. putida* genomes. The analysis was carried out using Artemis Comparison Tool and computed using TBLASTX with a cutoff E value of 1 E^-5^. The red bars between the DNA lines indicate individual TBLASTX matches, and the blue lines exhibit inverted matches. The cutoff identities and alignments length are 75% and 30 amino acids, respectively.

Comparisons of genome structure for UW4 vs completely sequenced *P. fluorescens, P. protegens,* and *P. putida* genomes are illustrated in Figure 5, with the red lines indicating individual TBLASTX matches and blue lines exhibiting inverted matches. The distribution of the genes among the *Pseudomonas* genomes showed that the unique genes are mostly located at the replication termini, whereas the orthologues are commonly present at the replication origin. The whole genome alignments showed extensive DNA rearrangement indicated by the blue lines, which is likely driven by repeat sequences within the genome. Moreover, the line plots revealed that genes in UW4 are more closely related to those in *P. fluorescens* and *P. protegens* than in *P. putida*, illustrated by the number of matches. This result is consistent with the results obtained from whole genome phylogenetic analysis.

### UW4 Taxonomy

**Figure 6:**
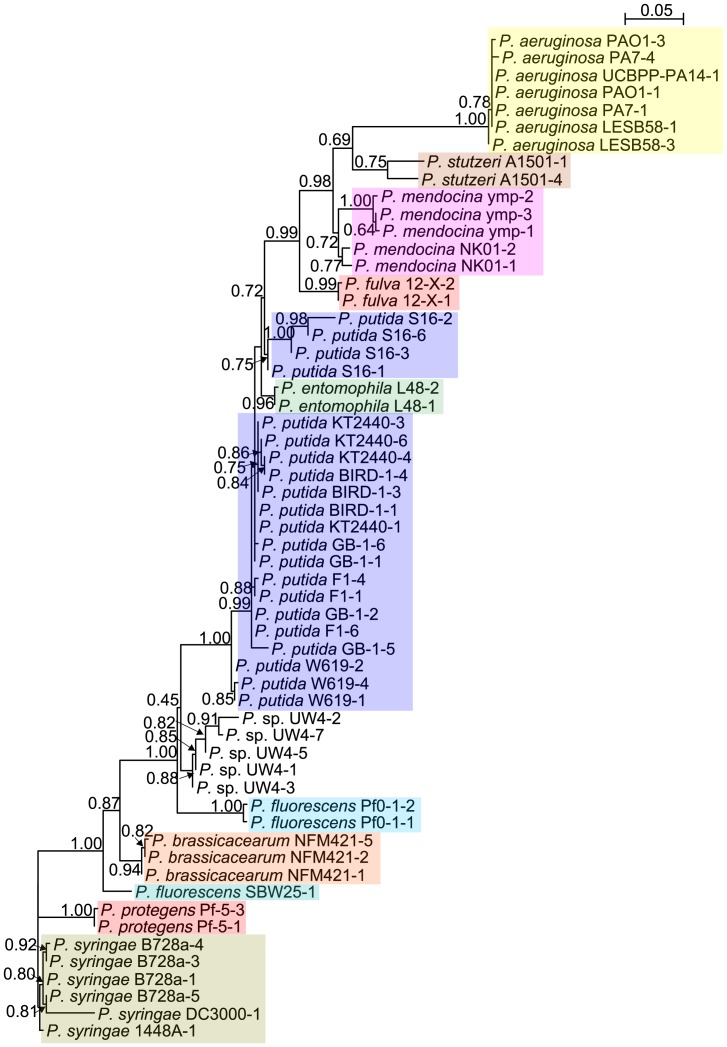
ML phylogenetic tree of 16S rRNA sequences from completely sequenced *Pseudomonas* genomes. Nodal support was evaluated by aLRT. Different species are shown in different colors. Only unique sequences from each genome were included for this analysis.

16S rRNA gene sequences are highly conserved among the same bacterial species and are frequently used to identify and classify microorganisms. It has been observed that the number of rRNA genes in prokaryotic genomes can vary from one to as many as 15 copies and the intragenic diversity ranges from 0.06% to 20.38% [122]. On the chromosome of UW4, seven ribosomal RNA (rrn) operons were identified. Among the seven 16S rRNA genes, three were found to have identical sequences (i.e., RNAs 3, 4 and 6). A ML phylogenetic tree was constructed for the unique 16S rRNA genes of *Pseudomonas* genomes (Figure 6). Although the 16S rRNA genes of UW4 are grouped with those of *P. putida*, the node support is only 0.45, indicating low confidence for the classification.

**Figure 7:**
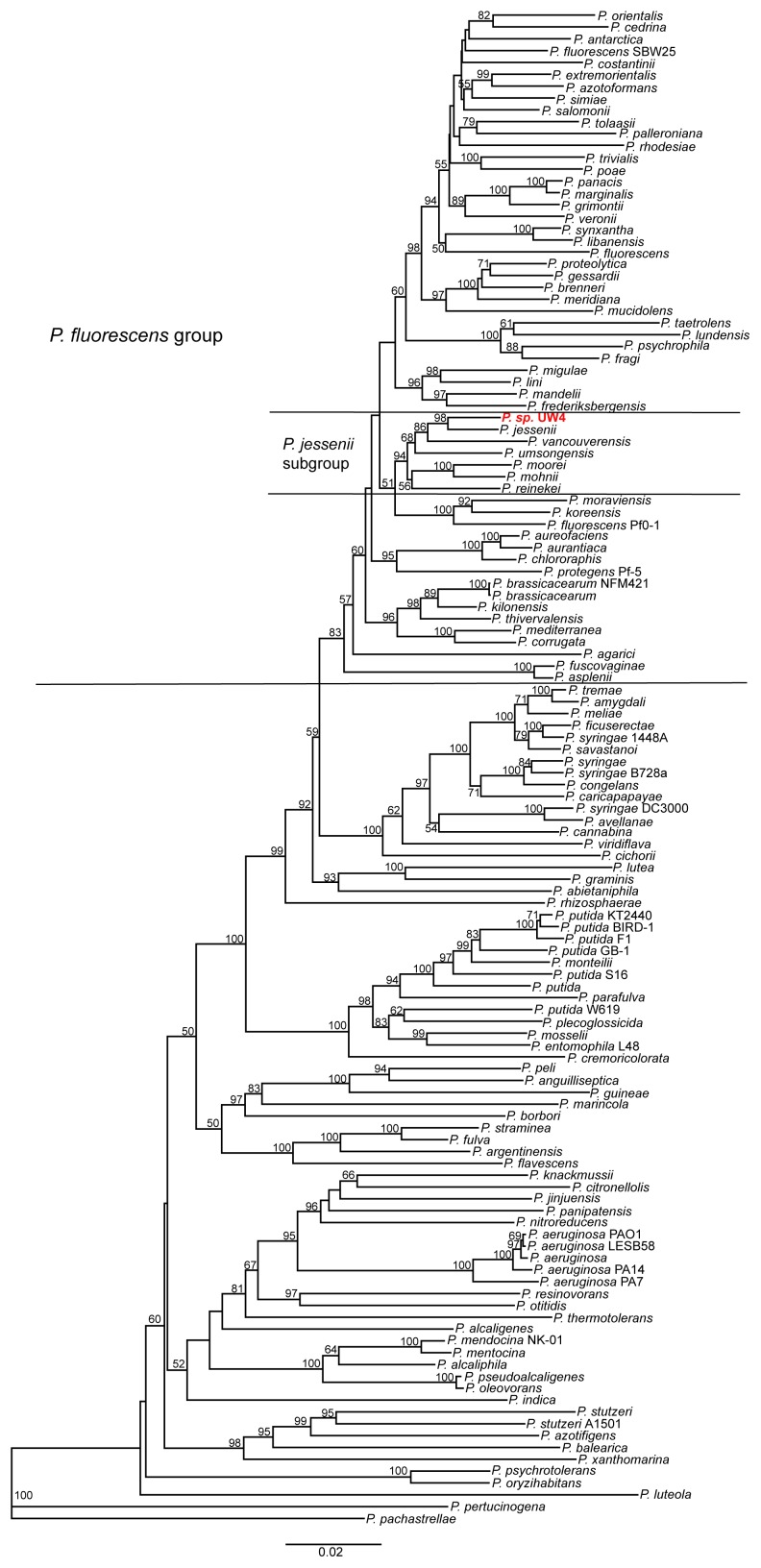
Phylogenetic tree of 128 *Pseudomonas* strains based on four concatenated genes including 16S rRNA, *gyrB*, *rpoB* and *rpoD*. Dendrogram was generated by neighbor-joining and distance matrix was calculated by the Jukes-Cantor algorithm. The bar at the bottom indicates sequence divergence. Nodal support was evaluated with 1000 bootstrap pseudoreplications and values of greater than 50% are shown at the nodes.

Additional analysis was conducted using the four concatenated housekeeping genes (16SrRNA, *gyrB*, *rpoD* and *rpoB*) of 128 *Pseudomonas* strains (Table 3 and Table S15) [123]. The phylogenetic tree revealed that UW4 fell into *P. fluorescens* group, *jessenii* subgroup (Figure 7). However, within the *jessenii* subgroup, the complete genome sequences of the other six species are not available.

Whole genome phylogenetic analysis suggested that UW4 is closer to *fluorescens* than to *putida*. However, 16S rRNA gene phylogeny of completely sequenced *Pseudomonas* genomes showed that UW4 is grouped with the *putida* clade, albeit with low confidence. Additional phylogenetic analysis of the four concatenated “housekeeping” genes showed that UW4 has a closer relationship with *P. jessenii*. These results raise the question of which species that UW4 belongs to.

*Pseudomonas* was first discovery by Migula in 1894. Since then, the taxonomy of *Pseudomonas* has always been controversial [124] and many *Pseudomonas* sp. have been reclassified as other species and/or genera through the years [125]–[140]. Although 16S rRNA gene is a basic tool of the current bacterial classification, it is known that very closely related species of bacteria cannot be differentiated based on this gene [141]–[147]. Therefore, many studies have shown that other genes, such as “housekeeping” genes *recA, atpD, carA, gyrB, rpoB*, *trpB*, should be used to assist bacterial species classification [139],[148]–[150]. Furthermore, the fact that most bacteria have multiple copies of 16S rRNA genes and their intragenomic diversities within individual genomes indicate that it is necessary to include all unique 16S rRNA genes of one bacterium for its identification. However, without knowing the complete genome sequence of the bacterium, one can hardly obtain all the sequences of its 16S rRNA genes.

Since the resolution of 16S rRNA tree was not sufficient to differentiate UW4 from other closely related *Pseudomonas* species, the classification of this bacterium should follow the whole genome phylogeny based on the conserved genes among all sequenced *Pseudomonas* genomes, as well as the phylogeny of the “housekeeping” genes, which indicated that it belongs to *P. fluorescens* group, *jessenii* subgroup. Furthermore, according to the Bergey’s Manual of Determinative Bacteriology [151], *P. fluorescens* is positive for nitrate reduction, while *P. putida* is negative. In the genome of UW4, the presence of a putative nitrate reductase (PputUW4_03644) supports the reclassification of UW4 into the *fluorescens* group.

### Conclusions

Genome sequencing of UW4 has opened up a number of opportunities to study this PGPB in the future, and knowledge of this sequence will benefit the development of a more complete understanding of the mechanisms used by this bacterium to promote plant growth. From the results of genome analyses, it was concluded that UW4 has a better fit within the *fluorescens* group rather than the *putida* group. Knowing the complete genome sequence of UW4 allows us to see this bacterium from a whole new point of view. Because biological functions rely on interactions between different biomolecules, rather than a single gene product, the availability of the whole genetic contents of this organism will surely help to provide more insight in unraveling the complex biological mechanisms that UW4 and other similar organisms use to promote plant growth. This work aims to initiate a more comprehensive study of the strain UW4. The analyses that have been done will provide a fundamental basis for future studies towards fully understanding the functioning of this organism.

## Materials and Methods

### Bacterial Growth and DNA Extraction

A single colony of *P.* sp. UW4 grown on Tryptic Soy agar (Difco Laboratories, Detroit, MI) was inoculated into 5 mL of TSB (Difco Laboratories, Detroit, MI) and grown overnight with shaking at 30°C. Bacterial cells were collected by centrifugation and the genomic DNA was extracted with a Wizard® Genomic DNA purification kit (Promega, Madison, WI, USA) according to the manufacturer’s instructions. *E. coli* DH5α [152] was used as a recipient for recombinant plasmids. This strain and its transformants with different plasmids were grown at 37°C in Luria-Bertani (LB) broth medium (Difco Laboratories, Detroit, MI), with appropriate antibiotics. Ampicillin was added at 100 µg/mL for *E. coli*.

### Whole Genome Sequencing and Assembly

The complete genomic sequencing was carried out at The McGill University and Genome Quebec Innovation Center where they used the current Roche GS-FLX Titanium chemistry protocols in place to sequence the genomic DNA. First, a shotgun library was prepared from 5 µg of DNA and subsequently sequenced, generating 203,178 reads in 74,063,913 bp of sequencing data. 93% of the reads were fully assembled into 312 large contigs, ranging from 518–197,691 bp. The sum of the large contigs’ size is 6,049,654 bp and about 12× of the sequencing coverage was achieved. In order to facilitate gap closure in the genome sequence, an 8 Kb paired-end library was then constructed using 15 µg of DNA to re-sequence the entire genome. After sequencing, 186,877 reads were generated in 73,775,344 bp of sequencing data. Combining the results of shotgun and paired-end sequencing, 96% of the reads were fully assembled into 122 large contigs ranging from 500–356,439 bp. Ten ordered and oriented scaffolds with a genome size of 6.22 Mb were obtained. Using paired-end sequencing, another 12× genome coverage was achieved. *De novo* sequence assembly was completed using Roche’s Newbler assembler v.2.0.01.14 at The McGill University and Genome Quebec Innovation Center. Gaps between the contigs were filled in by sequencing the PCR products using Applied Biosystems 3730xl DNA Analyzers at The McGill University and Genome Quebec Innovation Center, University of Guelph’s Advanced Analysis Centre, and York University Core Molecular Biology and DNA Sequencing Facility. Initially, 100 pairs of primers were designed to fill in the 100 gaps. Then, primer walking was used to close the gaps that were greater than 1.5 Kb. KOD Hot Start DNA Polymerase (EMD Millipore, MA, United States) and GoTaq® Hot Start Polymerase (Promega, WI, United States) were used for PCR amplification.

### Genome Annotation and Analysis

The *P.* sp. UW4 genome sequence was first annotated using web-based automated pipelines including Bacterial Annotation System (BASys) v1.0 [153] and Integrative Services for Genomic Analysis (ISGA) v1.2 [154]. Putative CDS were identified by Glimmer v3.02 [155]) and Prokaryotic Dynamic Programming Genefinding Algorithm (Prodigal) v2.5 [156]. The results from the two programs were combined and manually reviewed. Ribosomal RNA and transfer RNA genes were predicted by RNAmmer v1.2 [157] and tRNAScan-SE [158], which are embedded in the ISGA annotation pipeline. Next, functional annotation of the identified genes was conducted by a sequence similarity search against non-redundant (NR) protein database at the GenBank by BLAST, and putative function was assigned to each gene with a cutoff E-value of ≤1 E^−05^. Cluster of Orthologous Group (COG) and enzyme-coding genes were predicted by COG Finder 1.0 and ECNumber Finder in BASys. With the ISGA pipeline, COG was searched against the NCBI COG database [159]–[160] and an E.C. number was assigned by PRIAM [161]. The results from both pipelines were compared and manually corrected based on the current COG database and Enzyme nomenclature database [162]. Protein localization was predicted by PSORTb v3.0.2 [163] and genomic islands (GIs) were detected using IslandViewer [164]. Repeat sequences were examined by Tandem Repeats Finder v4.04 [165]. The metabolic pathways were constructed using Pathway Tools v15.5 [166] and the KEGG database [167]. Genome comparisons among 10 completely sequenced *P. putida* and *P. fluorescens* genomes were carried out using TBLASTX [168] and displayed by the Artemis Comparison Tool (ACT) [169]. Orthologs in the 21 *Pseudomonas* genomes were identified using Roundup [170] with the most stringent blast E-value (1 E^−20^) and divergence thresholds (0.2). Then the amino acid sequences of the core genes were aligned using the MUSCLE program in SeaView v4.3.2 [171]–[172], and poorly aligned regions were removed manually using Geneious Pro 5.4.6 [173]. Before constructing a maximum likelihood (ML) tree for each alignment, the model of protein evolution was selected using PROTTEST v2.4 [174]. Next, a ML tree was built using PHYML v3.0 [175] embedded in SeaView v4.3.2 with the appropriate model for each alignment. Nodal support was evaluated by the approximate likelihood ratio test (aLRT) [176]. Based on all the orthologs that were identified, a phylogenetic tree of 21 different *Pseudomonas* species was constructed using the consensus tree program of Geneious Pro 5.4.6 [173]. DNAPlotter [177] was used to draw a *P.* sp. UW4 genome atlas.

### Phylogeny of 16S rRNA Genes of *Pseudomonas* Genomes

The 16S rRNA gene sequences of *P.* sp. UW4 were aligned with those of the publicly available *Pseudomonas* genome sequences using the MUSCLE program in SeaView v4.3.2 [171]–[172] and refined manually using Geneious Pro v5.4.6 [173]. All the 21 *Pseudomonas* genomes have multiple copies of 16S rRNA genes, and only unique sequences were included in this analysis. The substitution model was selected using jModeltest v0.1.1 [178] and a ML tree was built by PHYML v3.0 [175] in SeaView v4.3.2 with a general time-reversible model (GTR), with the nodal support assessed by aLRT.

### Phylogeny of Four Concatenated “Housekeeping” Genes of *Pseudomonas* Strains

The concatenated sequences of 16S rRNA, *gyrB*, *rpoD* and *rpoB* of 128 *Pseudomonas* strains (Accession number shown in Table 3 and Table S15) were aligned using the MUSCLE program in SeaView v4.3.2 [171]–[172] and refined manually using Geneious Pro v5.4.6 [173]. The order of the four genes in the concatenated sequence is 16S rRNA, *gyrB*, *rpoD* and *rpoB*. A neighbor-joining tree was constructed using Jukes-Cantor algorithm [179]. Nodal support was evaluated with 1000 bootstrap pseudoreplications.

### Accession Numbers

The genomic sequences of *P.* sp. UW4 have been deposited in GenBank under accession number CP003880.

## Supporting Information

Figure S1 Pyoverdine synthesis genes in *P.* sp. UW4. Genes are not drawn to scale and are oriented according to the direction of transcription.
(TIF)

Figure S2 *P.* sp. UW4 predicted pyoverdine biosynthesis pathway.
(TIF)

Figure S3 *P.* sp. UW4 IAA biosynthesis pathways.
(TIF)

Table S1 *P.* sp. UW4 Pseudogenes.
(DOCX)

Table S2 Genomic Islands of *P.* sp. UW4 Predicted by IslandViewer.
(DOCX)

Table S3 *P*. sp. UW4 IS Elements.
(DOCX)

Table S4 *P.* sp. UW4 Phage Related CDSs.
(DOCX)

Table S5 Tandem Repeats Identified in *P.* sp. UW4.
(DOCX)

Table S6 Genes Associated with Pyoverdine Synthesis in *P.* sp. UW4.
(DOCX)

Table S7 *P.* sp. UW4 Multidrug Efflux Systems.
(DOCX)

Table S8 Genes potentially involved in metal resistance of *P.* sp, UW4.
(DOCX)

Table S9 Protein secretion systems in *P.* sp, UW4.
(DOCX)

Table S10 Type IV Pilus Genes in *P.* sp, UW4.
(DOCX)

Table S11 Putative Orthologous Relations Between UW4 and completely sequenced *Pseudomonas* genomes.
(DOCX)

Table S12 Predicted CDSs that share similarities with other *Pseudomonas* sp.
(DOCX)

Table S13 Putative unique CDSs in *P.* sp. UW4.
(DOCX)

Table S14 Predicted UW4 CDSs that share sequence similarities to those in other genera only.
(DOCX)

Table S15 GenBank accession numbers of the sequences used in the analysis of UW4 taxonomy.
(DOCX)
