# Supplementary figures and images for: The Complete Genome Sequence of the Plant Growth-Promoting Bacterium Pseudomonas sp. UW4
Source: PLoS One. 2013 Mar 13;8(3):e58640. doi: 10.1371/journal.pone.0058640 (PMC3596284; doi:10.1371/journal.pone.0058640)

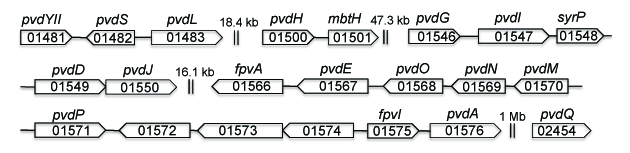

Supplement: Figure S1 — Pyoverdine synthesis genes in P. sp. UW4. Genes are not drawn to scale and are oriented according to the direction of transcription. (TIF) [file pone.0058640.s001.tif]

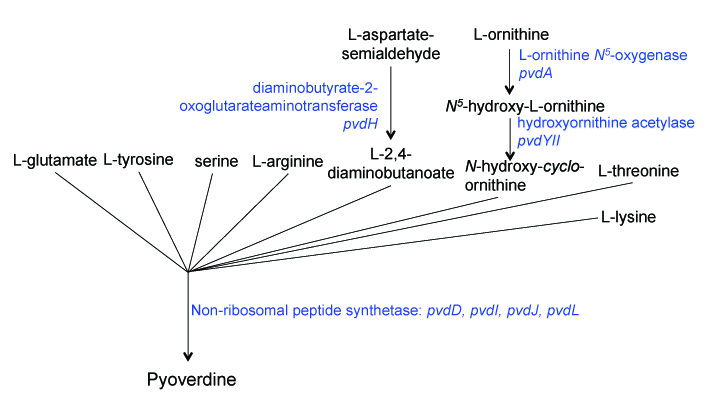

Supplement: Figure S2 — P. sp. UW4 predicted pyoverdine biosynthesis pathway. (TIF) [file pone.0058640.s002.tif]

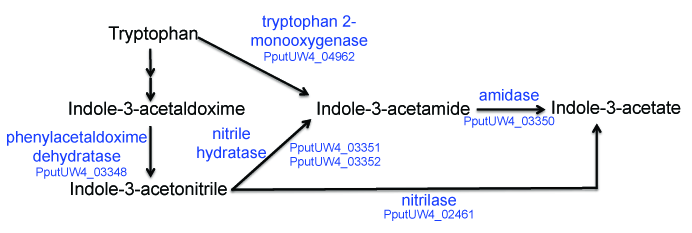

Supplement: Figure S3 — P. sp. UW4 IAA biosynthesis pathways. (TIF) [file pone.0058640.s003.tif]
