# Supplementary material for: The Complete Genome Sequence of the Plant Growth-Promoting Bacterium Pseudomonas sp. UW4
Source: PLoS One. 2013 Mar 13;8(3):e58640. doi: 10.1371/journal.pone.0058640 (PMC3596284; doi:10.1371/journal.pone.0058640)
Supplement: Table S1 — P. sp. UW4 Pseudogenes. (DOCX) [file pone.0058640.s004.docx]

Table S1. *Pseudomonas* sp. UW4 Pseudogenes

| locus_tag | product | pseudo |
| --- | --- | --- |
| PputUW4_00114 | von Willebrand factor, type A domain protein | frame shift |
| PputUW4_00636 | hypothetical protein | frame shift |
| PputUW4_01249 | methyltransferase | N-terminus missing fragment |
| PputUW4_01484 | transcriptional regulator | N- and C-terminus missing fragment |
| PputUW4_01956 | hypothetical protein | frame shift |
| PputUW4_02022 | ATP-dependent DNA ligase LigD | N-terminus missing fragment |
| PputUW4_02109 | long-chain fatty acid transporter | C-terminus missing fragment |
| PputUW4_02123 | IS30 family transposase | N-terminus missing fragment |
| PputUW4_02128 | IS1182 family transposase | frame shift |
| PputUW4_02138 | hypothetical protein | N- and C-terminus missing fragment |
| PputUW4_02368 | IclR family transcriptional regulator | N-terminus missing fragment |
| PputUW4_02422 | LysR family transcriptional regulator | N- and C-terminus missing fragment |
| PputUW4_02514 | xylulose kinase | N-terminus missing fragment |
| PputUW4_02516 | AraC family transcriptional regulator | C-terminus missing fragment |
| PputUW4_03356 | phage integrase | N-terminus missing fragment |
| PputUW4_03378 | LuxR family transcriptional regulator | N-terminus missing fragment |
| PputUW4_03546 | IS630 family transposase, truncated | C-terminus missing fragment |
| PputUW4_04659 | type IV pilus-associated protein | N-terminus missing fragment |
| PputUW4_04689 | glutamate dehydrogenase | N- and C-terminus missing fragment |
| PputUW4_05202 | glutamine amidotransferase | C-terminus missing fragment |
