# Supplementary material for: The Complete Genome Sequence of the Plant Growth-Promoting Bacterium Pseudomonas sp. UW4
Source: PLoS One. 2013 Mar 13;8(3):e58640. doi: 10.1371/journal.pone.0058640 (PMC3596284; doi:10.1371/journal.pone.0058640)
Supplement: Table S2 — Genomic Islands of P. sp. UW4 Predicted by IslandViewer. (DOCX) [file pone.0058640.s005.docx]

Table S2. Genomic Islands of *Pseudomonas* sp. UW4 Predicted by IslandViewer

| Genomic Islands | | | | Genes | | GC content |
| --- | --- | --- | --- | --- | --- | --- |
|  | Start | End | Size (bp) | Locus ID | Product |  |
| 1 | 743,662 | 749,401 | 5,740 | PputUW4_00639 | McrBC 5-methylcytosine restriction system component-like protein | 53.94 |
|  |  |  |  | PputUW4_00640 | hypothetical protein |  |
|  |  |  |  | PputUW4_00641 | hypothetical protein |  |
|  |  |  |  | PputUW4_00642 | hypothetical protein |  |
|  |  |  |  | PputUW4_00643 | hypothetical protein |  |
|  |  |  |  | PputUW4_00644 | hypothetical protein |  |
|  |  |  |  | PputUW4_00645 | hypothetical protein |  |
| 2 | 780,455 | 791,108 | 10,654 | PputUW4_00663 | hypothetical protein | 55.06 |
|  |  |  |  | PputUW4_00664 | putative helicase |  |
|  |  |  |  | PputUW4_00665 | DNA helicase |  |
|  |  |  |  | PputUW4_00666 | hypothetical protein |  |
| 3 | 797,778 | 809,172 | 11,395 | PputUW4_00670 | chemotaxis protein MotB-related protein | 54.74 |
|  |  |  |  | PputUW4_00671 | hypothetical protein |  |
|  |  |  |  | PputUW4_00672 | SNF2 family DNA/RNA helicase |  |
|  |  |  |  | PputUW4_00673 | 5-methylcytosine-specific restriction protein C |  |
|  |  |  |  | PputUW4_00674 | hypothetical protein |  |
|  |  |  |  | PputUW4_00675 | Phage integrase |  |
| 4 | 1,078,659 | 1,088,425 | 9,767 | PputUW4_00914 | NAD-dependent formate dehydrogenase gamma subunit | 52.94 |
|  |  |  |  | PputUW4_00915 | type III restriction protein res subunit |  |
|  |  |  |  | PputUW4_00916 | hypothetical protein |  |
| 5 | 1,598,659 | 1,609,449 | 10,791 | PputUW4_01365 | putative lipoprotein | 44.61 |
|  |  |  |  | PputUW4_01366 | Integration host factor subunit beta |  |
|  |  |  |  | PputUW4_01367 | chain length determinant family protein |  |
|  |  |  |  | PputUW4_01368 | acetyltransferase |  |
|  |  |  |  | PputUW4_01369 | lipopolysaccharide biosynthesis protein RffA |  |
|  |  |  |  | PputUW4_01370 | hypothetical protein |  |
|  |  |  |  | PputUW4_01371 | hypothetical protein |  |
|  |  |  |  | PputUW4_01372 | hypothetical protein |  |
|  |  |  |  | PputUW4_01373 | hypothetical protein |  |
|  |  |  |  | PputUW4_01374 | glycosyl transferases group 1 family protein |  |
| 6 | 2,436,768 | 2,446,210 | 9,443 | PputUW4_02091 | hypothetical protein | 54.74 |
|  |  |  |  | PputUW4_02092 | hypothetical protein |  |
|  |  |  |  | PputUW4_02093 | hypothetical protein |  |
|  |  |  |  | PputUW4_02094 | hypothetical protein |  |
|  |  |  |  | PputUW4_02095 | diguanylate cyclase |  |
|  |  |  |  | PputUW4_02096 | hypothetical protein |  |
|  |  |  |  | PputUW4_02097 | acetyltransferase |  |
|  |  |  |  | PputUW4_02098 | MarR family transcriptional regulator |  |
|  |  |  |  | PputUW4_02099 | AraC family transcriptional regulator |  |
| 7 | 2,464,903 | 2,490,567 | 25,665 | PputUW4_02114 | quinone oxidoreductase | 55.11 |
|  |  |  |  | PputUW4_02115 | methionine sulfoxide reductase A |  |
|  |  |  |  | PputUW4_02116 | RND family efflux transporter, MFP subunit |  |
|  |  |  |  | PputUW4_02117 | ABC transporter family protein |  |
|  |  |  |  | PputUW4_02118 | peptide ABC transporter permease |  |
|  |  |  |  | PputUW4_02119 | peptide ABC transporter permease |  |
|  |  |  |  | PputUW4_02120 | IS1182 family transposase |  |
|  |  |  |  | PputUW4_02121 | ThiF family protein |  |
|  |  |  |  | PputUW4_02122 | hypothetical protein |  |
|  |  |  |  | PputUW4_02123 | hypothetical protein |  |
|  |  |  |  | PputUW4_02124 | hypothetical protein |  |
|  |  |  |  | PputUW4_02125 | hypothetical protein |  |
|  |  |  |  | PputUW4_02126 | hypothetical protein |  |
|  |  |  |  | PputUW4_02127 | IS1182 family transposase, truncated |  |
|  |  |  |  | PputUW4_02128 | IS1182 family transposase, truncated |  |
|  |  |  |  | PputUW4_02129 | hypothetical protein |  |
|  |  |  |  | PputUW4_02130 | hypothetical protein |  |
|  |  |  |  | PputUW4_02131 | IS1182 family transposase |  |
|  |  |  |  | PputUW4_02132 | polysaccharide deacetylase family protein |  |
|  |  |  |  | PputUW4_02133 | GCN5-related N-acetyltransferase |  |
|  |  |  |  | PputUW4_02134 | hypothetical protein |  |
|  |  |  |  | PputUW4_02135 | HxlR family transcriptional regulator |  |
|  |  |  |  | PputUW4_02136 | nitrilase |  |
|  |  |  |  | PputUW4_02137 | glutathione-dependent formaldehyde-activatingGFA |  |
| 8 | 2,522,087 | 2,526,716 | 4,630 | PputUW4_02162 | hypothetical protein | 48.66 |
|  |  |  |  | PputUW4_02163 | integrase family protein |  |
|  |  |  |  | PputUW4_02164 | hypothetical protein |  |
|  |  |  |  | PputUW4_02165 | hypothetical protein |  |
|  |  |  |  | PputUW4_02166 | hypothetical protein |  |
| 9 | 2,593,656 | 2,597,985 | 4,330 | PputUW4_02214 | cytosine/purines uracil thiamine allantoinpermease | 55.66 |
|  |  |  |  | PputUW4_02215 | histone deacetylase |  |
|  |  |  |  | PputUW4_02216 | LysR family transcriptional regulator |  |
|  |  |  |  | PputUW4_02217 | lysine exporter protein LysE/YggA |  |
| 10 | 2,943,190 | 2,947,513 | 4,323 | PputUW4_02529 | hypothetical protein | 49.35 |
|  |  |  |  | PputUW4_02530 | hypothetical protein |  |
| 11 | 3,036,561 | 3,041,305 | 4,745 | PputUW4_02595 | 2,4-dihydroxyacetophenone dioxygenase | 63.92 |
|  |  |  |  | PputUW4_02596 | hypothetical protein |  |
|  |  |  |  | PputUW4_02597 | short chain dehydrogenase family protein |  |
|  |  |  |  | PputUW4_02598 | thiolase |  |
|  |  |  |  | PputUW4_02599 | AMP-dependent synthetase and ligase |  |
| 12 | 3,099,312 | 3,104,459 | 5,148 | PputUW4_02647 | LysR family transcriptional regulator | 53.17 |
|  |  |  |  | PputUW4_02648 | amino acid transporter |  |
|  |  |  |  | PputUW4_02649 | cystathionine gamma-synthase |  |
|  |  |  |  | PputUW4_02650 | AraC family transcriptional regulator |  |
| 13 | 3,176,210 | 3,186,955 | 10,746 | PputUW4_02722 | hypothetical protein | 53.82 |
|  |  |  |  | PputUW4_02723 | CadC family transcriptional regulator |  |
|  |  |  |  | PputUW4_02724 | hypothetical protein |  |
|  |  |  |  | PputUW4_02725 | hypothetical protein |  |
|  |  |  |  | PputUW4_02726 | AraC family transcriptional regulator |  |
|  |  |  |  | PputUW4_02727 | hypothetical protein |  |
|  |  |  |  | PputUW4_02728 | OmpA-like transmembrane domain protein |  |
|  |  |  |  | PputUW4_02729 | hypothetical protein |  |
| 14 | 3,737,337 | 3,743,674 | 6,338 | PputUW4_03190 | two component LuxR family transcriptional regulator | 58.82 |
|  |  |  |  | PputUW4_03191 | hypothetical protein |  |
|  |  |  |  | PputUW4_03192 | IS110 family transposase |  |
|  |  |  |  | PputUW4_03193 | XRE family transcriptional regulator |  |
|  |  |  |  | PputUW4_03194 | hypothetical protein |  |
|  |  |  |  | PputUW4_03195 | hypothetical protein |  |
|  |  |  |  | PputUW4_03196 | 3-oxoadipate enol-lactonase |  |
| 15 | 3,966,752 | 3,970,895 | 4,144 | PputUW4_03411 | hypothetical protein | 52.82 |
|  |  |  |  | PputUW4_03412 | hypothetical protein |  |
|  |  |  |  | PputUW4_03413 | hypothetical protein |  |
|  |  |  |  | PputUW4_03414 | hypothetical protein |  |
|  |  |  |  | PputUW4_03415 | flagella synthesis regulator FleN |  |
|  |  |  |  | PputUW4_03416 | hypothetical protein |  |
| 16 | 4,116,806 | 4,123,634 | 6,829 | PputUW4_03540 | glycerol-3-phosphate cytidyltransferase | 40.33 |
|  |  |  |  | PputUW4_03541 | polysaccharide biosynthesis protein |  |
|  |  |  |  | PputUW4_03542 | hypothetical protein |  |
|  |  |  |  | PputUW4_03543 | hypothetical protein |  |
|  |  |  |  | PputUW4_03544 | glucosyltransferase |  |
|  |  |  |  | PputUW4_03545 | glucosyltransferase |  |
|  |  |  |  | PputUW4_03546 | IS630 family transposase, truncated |  |
| 17 | 4,159,348 | 4,182,589 | 23,242 | PputUW4_03580 | integrase family protein | 54.4 |
|  |  |  |  | PputUW4_03581 | hypothetical protein |  |
|  |  |  |  | PputUW4_03582 | hypothetical protein |  |
|  |  |  |  | PputUW4_03583 | hypothetical protein |  |
|  |  |  |  | PputUW4_03584 | hypothetical protein |  |
|  |  |  |  | PputUW4_03585 | hypothetical protein |  |
|  |  |  |  | PputUW4_03586 | hypothetical protein |  |
|  |  |  |  | PputUW4_03587 | transposon resolvase |  |
|  |  |  |  | PputUW4_03588 | hypothetical protein |  |
|  |  |  |  | PputUW4_03589 | hypothetical protein |  |
|  |  |  |  | PputUW4_03590 | hypothetical protein |  |
|  |  |  |  | PputUW4_03591 | hypothetical protein |  |
|  |  |  |  | PputUW4_03592 | hypothetical protein |  |
|  |  |  |  | PputUW4_03593 | hypothetical protein |  |
|  |  |  |  | PputUW4_03594 | hypothetical protein |  |
|  |  |  |  | PputUW4_03595 | NAD-dependent DNA ligase LigA |  |
|  |  |  |  | PputUW4_03596 | cell division protein ZipA |  |
|  |  |  |  | PputUW4_03597 | chromosome partition protein |  |
|  |  |  |  | PputUW4_03598 | GntR family transcriptional regulator |  |
|  |  |  |  | PputUW4_03599 | xanthine dehydrogenase small subunit |  |
| 18 | 4,818,892 | 4,826,804 | 7,913 | PputUW4_04187 | hypothetical protein | 44.48 |
|  |  |  |  | PputUW4_04188 | cytosine-specific methyltransferase |  |
|  |  |  |  | PputUW4_04189 | hypothetical protein |  |
|  |  |  |  | PputUW4_04190 | histidine kinase |  |
|  |  |  |  | PputUW4_04191 | hypothetical protein |  |
|  |  |  |  | PputUW4_04192 | 5-methylcytosine-specific restriction enzyme |  |
|  |  |  |  | PputUW4_04193 | DNA mismatch endonuclease Vsr |  |
| 19 | 5,281,019 | 5,288,766 | 7,748 | PputUW4_04606 | paraquat-inducible protein A | 52.04 |
|  |  |  |  | PputUW4_04607 | paraquat-inducible protein A |  |
|  |  |  |  | PputUW4_04608 | sulfite oxidase subunit YedZ |  |
