# Supplementary material for: The Complete Genome Sequence of the Plant Growth-Promoting Bacterium Pseudomonas sp. UW4
Source: PLoS One. 2013 Mar 13;8(3):e58640. doi: 10.1371/journal.pone.0058640 (PMC3596284; doi:10.1371/journal.pone.0058640)
Supplement: Table S3 — P. sp. UW4 IS Elements. (DOCX) [file pone.0058640.s006.docx]

Table S3. *Pseudomonas* sp. UW4 IS Elements

| Family/Group | *P.* sp*.* UW4 CDS ID |
| --- | --- |
| IS*110* | PputUW4_00472 PputUW4_00838 PputUW4_02021 PputUW4_04586 PputUW4_05358 |
| IS*110*/IS*1111* | PputUW4_00316 PputUW4_01251 PputUW4_01993 PputUW4_03127 PputUW4_03192 PputUW4_03446 PputUW4_04223 PputUW4_04440 PputUW4_05131 PputUW4_05207 PputUW4_05330 |
| IS*1182* | PputUW4_01557 PputUW4_02120 PputUW4_02131 PputUW4_02159 PputUW4_02161 PputUW4_02210 PputUW4_02218 |
| IS*3*/IS*3* | PputUW4_00149 PputUW4_01499 PputUW4_01772 PputUW4_02011 PputUW4_03138 PputUW4_03920 PputUW4_04236 PputUW4_04344 |
| *Truncated* |  |
| IS*110*/IS*1111* | PputUW4_02996 |
| IS*1182* | PputUW4_02128 |
| IS*30* | PputUW4_02123 |
| IS*5*/IS*427* | PputUW4_03355 |
| IS*630* | PputUW4_03546 |
