# Supplementary material for: The Complete Genome Sequence of the Plant Growth-Promoting Bacterium Pseudomonas sp. UW4
Source: PLoS One. 2013 Mar 13;8(3):e58640. doi: 10.1371/journal.pone.0058640 (PMC3596284; doi:10.1371/journal.pone.0058640)
Supplement: Table S4 — P. sp. UW4 Phage Related CDSs. (DOCX) [file pone.0058640.s007.docx]

Table S4. *Pseudomonas* sp. UW4 Phage Related CDSs

| PputUW4_ | product |
| --- | --- |
| 00454 | HflK protein |
| 00455 | HflC protein |
| 00667 | phage integrase |
| 00675 | phage integrase |
| 01005 | GtrA family protein |
| 01006 | bactoprenol glucosyl transferase |
| 01026 | hypothetical protein |
| 01122 | phage repressor |
| 01555 | phage integrase family site specific recombinase |
| 01761 | phage repressor |
| 01772 | integrase catalytic protein |
| 02414 | cointegrate resolution protein T |
| 02415 | cointegrate resolution protein S |
| 02776 | phage integrase |
| 03264 | hypothetical protein |
| 03356 | phage integrase |
| 03587 | transposon resolvase |
| 04191 | hypothetical protein |
| 05296 | prophage antirepressor |
