# Supplementary material for: The Complete Genome Sequence of the Plant Growth-Promoting Bacterium Pseudomonas sp. UW4
Source: PLoS One. 2013 Mar 13;8(3):e58640. doi: 10.1371/journal.pone.0058640 (PMC3596284; doi:10.1371/journal.pone.0058640)
Supplement: Table S5 — Tandem Repeats Identified in P. sp. UW4. (DOCX) [file pone.0058640.s008.docx]

Table S5. Tandem Repeats Identified in *Pseudomonas* sp*.* UW4

| Start | End | Period Size | Consensus Size | Copy Number | Affected Gene or Region | Gene Product | Repeats Relative to CDS |
| --- | --- | --- | --- | --- | --- | --- | --- |
| 43924 | 43964 | 21 | 22 | 1.9 | 00041 | oligopeptidase A | inside |
| 121176 | 121255 | 27 | 28 | 3 | 00114 (pseudo) | von Willebrand factor, type A | inside |
| 121830 | 121859 | 6 | 6 | 5 |  |  |  |
| 124695 | 129038 | 318 | 317 | 13.7 |  |  |  |
| 129077 | 129901 | 300 | 300 | 2.8 |  |  |  |
| 130252 | 130291 | 6 | 6 | 6.7 |  |  |  |
| 448990 | 449029 | 21 | 21 | 1.9 | 00394 | dihydrolipoamide acetyltransferase | inside |
| 571073 | 571412 | 84 | 84 | 4 | 00495 | hypothetical protein | inside |
| 634896 | 634947 | 15 | 14 | 3.7 | 00552 | acetyl-CoA carboxylase biotin carboxyl carrier protein subunit | inside |
| 634923 | 634952 | 6 | 6 | 5 |  |  |  |
| 1058842 | 1059321 | 123 | 123 | 3.9 | 00900 | hypothetical protein | inside |
| 1237244 | 1237308 | 21 | 20 | 3.1 | 01040 | methyl-accepting chemotaxis protein | inside |
| 1373898 | 1373930 | 16 | 17 | 2 | 01167 | hypothetical protein | inside |
| 1408421 | 1408452 | 12 | 12 | 2.7 | 01195 | hypothetical protein | inside |
| 1479472 | 1479511 | 15 | 15 | 2.7 | 01263 | hypothetical protein | inside |
| 1479931 | 1480007 | 36 | 36 | 2.1 | 01264 | hypothetical protein | inside |
| 1807605 | 1807634 | 15 | 16 | 1.9 | 01547 | Non-ribosomal peptide synthetase | inside |
| 1865671 | 1865696 | 12 | 12 | 2.2 | 01581 | Hpt sensor hybrid histidine kinase | inside |
| 1920541 | 1921009 | 39 | 39 | 12 | 01627 | hypothetical protein | inside |
| 1939911 | 1939955 | 21 | 21 | 2.1 | 01646 | hypothetical protein | inside |
| 1940032 | 1940078 | 24 | 24 | 2 |  |  |  |
| 2029994 | 2030045 | 27 | 27 | 1.9 | 01722 | heme peroxidase | inside |
| 2030037 | 2030826 | 273 | 272 | 2.9 |  |  |  |
| 2030243 | 2030299 | 27 | 27 | 2.1 |  |  |  |
| 2030533 | 2030592 | 27 | 27 | 2.2 |  |  |  |
| 2067404 | 2067444 | 20 | 21 | 2 | 01753 | AFG1-like ATPase | inside |
| 2222958 | 2223231 | 39 | 39 | 7 | 01896 | hypothetical protein | inside |
| 2248389 | 2248418 | 15 | 15 | 2 | 01918 | hypothetical protein | inside |
| 2248428 | 2248469 | 15 | 15 | 2.8 |  |  |  |
| 2248458 | 2248521 | 30 | 30 | 2.2 |  |  |  |
| 2259311 | 2259560 | 84 | 84 | 3 | 01929 | hypothetical protein | inside |
| 2449938 | 2450234 | 33 | 33 | 9 | 02102 | hypothetical protein | inside |
| 2450214 | 2450295 | 18 | 18 | 4.6 |  |  |  |
| 2450218 | 2450303 | 36 | 36 | 2.4 |  |  |  |
| 2532903 | 2532935 | 6 | 6 | 5.3 | 02168 | cyclic beta 1-2 glucan synthetase | inside |
| 2544850 | 2544899 | 18 | 19 | 2.8 | 02176 | hypothetical protein | inside |
| 2544895 | 2544959 | 9 | 9 | 7.2 | 02176 | hypothetical protein | inside |
| 2562691 | 2563936 | 276 | 275 | 4.5 | 02185 | sensor histidine kinase | inside |
| 2600012 | 2600043 | 16 | 17 | 1.9 | 02219 | RipR family transcriptional regulator | inside |
| 2665103 | 2665175 | 27 | 27 | 2.7 | 02279 | hemolysin-type calcium-binding region | inside |
| 2665366 | 2665503 | 54 | 54 | 2.6 |  |  |  |
| 2665925 | 2665996 | 27 | 27 | 2.7 |  |  |  |
| 2682575 | 2682603 | 14 | 15 | 2 | 02296 | 3-oxoacyl-[acyl-carrier-protein] reductase | inside |
| 2801188 | 2801654 | 123 | 123 | 3.8 | 02390 | hypothetical protein | inside |
| 2975571 | 2975623 | 24 | 24 | 2.2 | 02551 | hypothetical protein | inside |
| 2975578 | 2975624 | 24 | 24 | 2 |  |  |  |
| 3112030 | 3112056 | 12 | 12 | 2.2 | 02659 | cation efflux family protein | inside |
| 3144781 | 3144819 | 18 | 17 | 2.4 | 02687 | hypothetical protein | inside |
| 3154032 | 3154065 | 15 | 15 | 2.3 | 02696 | hypothetical protein | inside |
| 3176471 | 3176601 | 67 | 68 | 1.9 | 02722 | hypothetical protein | inside |
| 3510124 | 3510909 | 300 | 300 | 2.6 | 02988 | hypothetical protein | inside |
| 3511360 | 3512139 | 375 | 375 | 2.1 |  |  |  |
| 3641058 | 3641162 | 48 | 48 | 2.2 | 03096 | hypothetical protein | inside |
| 3641142 | 3641185 | 18 | 18 | 2.4 |  |  |  |
| 3644694 | 3645032 | 126 | 126 | 2.7 | 03101 | hypothetical protein | inside |
| 3644757 | 3644959 | 63 | 63 | 3.2 |  |  |  |
| 3738308 | 3738659 | 78 | 78 | 4.5 | 03191 | hypothetical protein | inside |
| 3741377 | 3741429 | 27 | 27 | 2 | 03194 | hypothetical protein | inside |
| 3741468 | 3741516 | 24 | 24 | 2 |  |  |  |
| 3846098 | 3846150 | 15 | 15 | 3.5 | 03299 | hypothetical protein | inside |
| 3846136 | 3846204 | 18 | 18 | 3.7 |  |  |  |
| 3846167 | 3846212 | 21 | 21 | 2.2 |  |  |  |
| 3846236 | 3846265 | 9 | 9 | 3.3 |  |  |  |
| 3846422 | 3846586 | 36 | 36 | 4.6 |  |  |  |
| 4050739 | 4050793 | 24 | 24 | 2.3 | 03484 | copper-resistance protein CopA | inside |
| 4051392 | 4051496 | 39 | 39 | 2.7 | 03485 | copper resistance protein B | inside |
| 4155899 | 4155941 | 21 | 21 | 2 | 03577 | hypothetical protein | inside |
| 4165503 | 4165535 | 15 | 16 | 2.1 | 03584 | hypothetical protein | inside |
| 4216459 | 4216692 | 63 | 63 | 3.7 | 03630 | hypothetical protein | inside |
| 4394420 | 4394470 | 18 | 18 | 2.8 | 03800 | dihydrolipoamide succinyltransferase | inside |
| 4421694 | 4421724 | 15 | 15 | 2.1 | 03824 | urea transporter | inside |
| 4449929 | 4449975 | 15 | 15 | 3.1 | 03855 | chemotaxis sensor histidine kinase CheA | inside |
| 4469374 | 4469413 | 12 | 12 | 3.3 | 03876 | flagellar assembly protein H | inside |
| 4531905 | 4531945 | 21 | 22 | 1.9 | 03934 | TrkH family potassium uptake protein | inside |
| 4560584 | 4560618 | 18 | 18 | 2 | 03952 | ABC transporter, transmembrane region | inside |
| 4654006 | 4654046 | 18 | 18 | 2.3 | 04042 | ribonuclease E | inside |
| 4654015 | 4654049 | 18 | 18 | 1.9 |  |  |  |
| 4654106 | 4654169 | 27 | 27 | 2.4 |  |  |  |
| 4718832 | 4718880 | 21 | 21 | 2.2 | 04105 | GTP diphosphokinase | inside |
| 4767521 | 4769427 | 462 | 462 | 4.1 | 04149 | hypothetical protein | inside |
| 4769292 | 4769562 | 135 | 135 | 2 |  |  |  |
| 4769427 | 4770439 | 462 | 463 | 2.2 |  |  |  |
| 4785020 | 4785089 | 34 | 34 | 2.1 | 04161 | hypothetical protein | inside |
| 4850751 | 4850780 | 15 | 15 | 2 | 04221 | lysine exporter protein LysE/YggA | inside |
| 4930376 | 4930450 | 27 | 24 | 3.1 | 04292 | TolA colicin import membrane protein | inside |
| 4930413 | 4930507 | 24 | 25 | 3.8 |  |  |  |
| 4930462 | 4930537 | 24 | 24 | 3.2 |  |  |  |
| 4930518 | 4930582 | 24 | 24 | 2.7 |  |  |  |
| 4930537 | 4930591 | 24 | 21 | 2.5 |  |  |  |
| 4930623 | 4930735 | 18 | 18 | 6.3 |  |  |  |
| 5038760 | 5038790 | 9 | 9 | 3.4 | 04386 | chaperonin GroEL | inside |
| 5316884 | 5316909 | 12 | 12 | 2.2 | 04634 | glucose-6-phosphate isomerase | inside |
| 5439683 | 5439712 | 15 | 15 | 2 | 04751 | hypothetical protein | inside |
| 5439985 | 5440013 | 12 | 12 | 2.4 |  |  |  |
| 5550454 | 5550510 | 27 | 27 | 2.1 | 04853 | single-stranded DNA-binding protein | inside |
| 5727150 | 5727183 | 12 | 12 | 2.8 | 05015 | hypothetical protein | inside |
| 5797511 | 5797551 | 21 | 21 | 2 | 05076 | formate/nitrate transporter | inside |
| 5803893 | 5803923 | 12 | 12 | 2.6 | 05081 | ATP-dependent RNA helicase | inside |
| 5821054 | 5821090 | 12 | 12 | 3.1 | 05100 | signal transducer, CheW | inside |
| 5864843 | 5864867 | 12 | 12 | 2.1 | 05143 | aldehyde dehydrogenase | inside |
| 5914851 | 5915078 | 48 | 48 | 4.8 | 05191 | S-type Pyocin | inside |
| 5919408 | 5919444 | 15 | 16 | 2.5 | 05193 | AraC family transcriptional regulator | inside |
| 5971992 | 5972025 | 15 | 16 | 2.2 | 05244 | TonB domain-containing protein | inside |
| 5997825 | 5997883 | 30 | 30 | 2 | 05268 | hypothetical protein | inside |
| 5997897 | 5998087 | 24 | 24 | 7.7 |  |  |  |
| 6033004 | 6033039 | 18 | 18 | 2 | 05304 | xanthine phosphoribosyltransferase | inside |
| 6069812 | 6069848 | 15 | 16 | 2.4 | 05342 | TonB-like protein | inside |
| 1948571 | 1948892 | 133 | 134 | 2.4 | 01657 | hypothetical protein | inside and downstream |
| 3574756 | 3575299 | 260 | 260 | 2.1 | 03043 | hypothetical protein | inside and downstream |
| 3768552 | 3768922 | 123 | 123 | 3 | 03221 | hypothetical protein | inside and downstream |
| 3956354 | 3956411 | 17 | 17 | 3.5 | 03399 | LacI family transcription regulator | inside and downstream |
| 4134951 | 4135470 | 284 | 284 | 1.8 | 03556 | aerotaxis receptor | inside and downstream |
| 4785054 | 4785456 | 123 | 123 | 3.3 | 04161 | hypothetical protein | inside and downstream |
| 1954356 | 1955116 | 263 | 265 | 2.9 | 01661 | hypothetical protein | inside and upstream |
| 3156644 | 3157707 | 123 | 123 | 8.7 | 02700 | hypothetical protein | inside and upstream |
| 3176187 | 3176540 | 123 | 123 | 2.9 | 02722 | hypothetical protein | inside and upstream |
| 3235498 | 3235719 | 123 | 123 | 1.8 |  |  |  |
| 3593725 | 3594335 | 136 | 136 | 4.5 | 03059 | hypothetical protein | inside and upstream |
| 3680599 | 3680921 | 133 | 133 | 2.4 | 03137 | hypothetical protein | inside and upstream |
| 4046312 | 4046894 | 123 | 123 | 4.7 | 03481 | hypothetical protein | inside and upstream |
| 3729837 | 3730254 | 133 | 133 | 3.1 | 03184 | hypothetical protein | inside, upstream and downstream |
| 4858915 | 4859409 | 112 | 112 | 4.4 | 04229 | hypothetical protein | inside, upstream and downstream |
| 305869 | 306071 | 79 | 80 | 2.5 | intergenic region |  |  |
| 1002063 | 1002092 | 14 | 14 | 2.1 | intergenic region |  |  |
| 1374095 | 1374169 | 19 | 19 | 3.9 | intergenic region |  |  |
| 1824758 | 1824840 | 18 | 18 | 4.5 | intergenic region |  |  |
| 1824936 | 1824975 | 19 | 19 | 2.1 | intergenic region |  |  |
| 1916677 | 1916736 | 6 | 6 | 10 | intergenic region |  |  |
| 2176903 | 2177229 | 129 | 131 | 2.5 | intergenic region |  |  |
| 2187851 | 2187894 | 17 | 17 | 2.6 | intergenic region |  |  |
| 2196382 | 2196412 | 14 | 14 | 2.2 | intergenic region |  |  |
| 2229075 | 2229120 | 15 | 15 | 3 | intergenic region |  |  |
| 2354303 | 2354650 | 134 | 134 | 2.6 | intergenic region |  |  |
| 2355824 | 2356096 | 135 | 134 | 2 | intergenic region |  |  |
| 2375568 | 2375860 | 131 | 132 | 2.2 | intergenic region |  |  |
| 2388278 | 2388645 | 133 | 132 | 2.8 | intergenic region |  |  |
| 2436141 | 2436172 | 15 | 15 | 2.1 | intergenic region |  |  |
| 2450520 | 2450623 | 33 | 33 | 3.2 | intergenic region |  |  |
| 2471329 | 2471355 | 6 | 6 | 4.5 | intergenic region |  |  |
| 2546335 | 2546418 | 18 | 18 | 4.7 | intergenic region |  |  |
| 2546336 | 2546400 | 21 | 21 | 3.1 | intergenic region |  |  |
| 2546351 | 2546388 | 15 | 15 | 2.5 | intergenic region |  |  |
| 2546683 | 2546763 | 9 | 9 | 9 | intergenic region |  |  |
| 2546786 | 2546882 | 33 | 33 | 2.9 | intergenic region |  |  |
| 2546929 | 2547042 | 33 | 33 | 3.5 | intergenic region |  |  |
| 2546930 | 2547092 | 66 | 66 | 2.5 | intergenic region |  |  |
| 2546944 | 2546985 | 21 | 21 | 2 | intergenic region |  |  |
| 2547120 | 2547179 | 18 | 18 | 3.3 | intergenic region |  |  |
| 2565588 | 2565646 | 11 | 11 | 5.4 | intergenic region |  |  |
| 2646854 | 2647217 | 123 | 123 | 3 | intergenic region |  |  |
| 2707912 | 2707959 | 16 | 16 | 2.9 | intergenic region |  |  |
| 2744769 | 2744999 | 123 | 123 | 1.9 | intergenic region |  |  |
| 2759436 | 2759466 | 15 | 14 | 2.1 | intergenic region |  |  |
| 2888440 | 2888575 | 68 | 68 | 2 | intergenic region |  |  |
| 2994262 | 2994913 | 123 | 123 | 5.3 | intergenic region |  |  |
| 3022913 | 3023148 | 125 | 125 | 1.9 | intergenic region |  |  |
| 3110014 | 3110072 | 8 | 8 | 7.4 | intergenic region |  |  |
| 3129909 | 3129952 | 15 | 15 | 2.9 | intergenic region |  |  |
| 3138106 | 3138445 | 123 | 123 | 2.8 | intergenic region |  |  |
| 3279148 | 3279185 | 19 | 19 | 2 | intergenic region |  |  |
| 3354637 | 3354674 | 19 | 19 | 2 | intergenic region |  |  |
| 3408820 | 3408861 | 18 | 18 | 2.3 | intergenic region |  |  |
| 3421369 | 3422102 | 123 | 123 | 5.9 | intergenic region |  |  |
| 3432149 | 3432476 | 123 | 123 | 2.7 | intergenic region |  |  |
| 3577587 | 3577730 | 68 | 68 | 2.1 | intergenic region |  |  |
| 3577672 | 3578137 | 123 | 123 | 3.8 | intergenic region |  |  |
| 3591390 | 3591761 | 125 | 125 | 3 | intergenic region |  |  |
| 3654879 | 3654911 | 17 | 17 | 1.9 | intergenic region |  |  |
| 3655013 | 3655045 | 17 | 17 | 1.9 | intergenic region |  |  |
| 3671332 | 3671585 | 123 | 123 | 2.1 | intergenic region |  |  |
| 3698756 | 3699197 | 169 | 167 | 2.6 | intergenic region |  |  |
| 3751285 | 3751347 | 19 | 19 | 3.4 | intergenic region |  |  |
| 3756009 | 3756048 | 19 | 19 | 2.1 | intergenic region |  |  |
| 3784412 | 3784469 | 28 | 28 | 2.1 | intergenic region |  |  |
| 4128189 | 4128229 | 18 | 18 | 2.3 | intergenic region |  |  |
| 4731531 | 4731562 | 16 | 16 | 2 | intergenic region |  |  |
| 4764716 | 4764748 | 17 | 17 | 1.9 | intergenic region |  |  |
| 4772590 | 4772615 | 9 | 9 | 2.9 | intergenic region |  |  |
| 5015287 | 5015322 | 18 | 18 | 2 | intergenic region |  |  |
| 5262394 | 5262754 | 141 | 141 | 2.6 | intergenic region |  |  |
| 5755701 | 5755743 | 21 | 21 | 2 | intergenic region |  |  |
| 6127819 | 6127848 | 14 | 14 | 2.1 | intergenic region |  |  |
