# Supplementary material for: The Complete Genome Sequence of the Plant Growth-Promoting Bacterium Pseudomonas sp. UW4
Source: PLoS One. 2013 Mar 13;8(3):e58640. doi: 10.1371/journal.pone.0058640 (PMC3596284; doi:10.1371/journal.pone.0058640)
Supplement: Table S6 — Genes Associated with Pyoverdine Synthesis in P. sp. UW4. (DOCX) [file pone.0058640.s009.docx]

Table S6. Genes associated with pyoverdine synthesis in *Pseudomonas* sp*.* UW4.

| CDS ID | Gene | Function |
| --- | --- | --- |
| 01481 | *pvdYII* | Hydroxyornithine acetylase |
| 01482 | *pvdS* | ECF iron sigma factor |
| 01483 | *pvdL* | Non-ribosomal peptide synthetase |
| 01500 | *pvdH* | Aminotransferase |
| 01501 | *mbtH* | Unknown |
| 01546 | *pvdG* | Thioesterase |
| 01547 | *pvdI* | Non-ribosomal peptide synthetase |
| 01548 | *syrP* | Pyoverdine biosynthesis regulatory protein |
| 01549 | *pvdD* | Non-ribosomal peptide synthetase |
| 01550 | *pvdJ* | Non-ribosomal peptide synthetase |
| 01566 | *fpvA* | TonB-dependent pyoverdine receptor |
| 01567 | *pvdE* | ABC transporter |
| 01568 | *pvdO* | Unknown |
| 01569 | *pvdN* | Aminotransferase |
| 01570 | *pvdM* | Dipeptidase |
| 01571 | *pvdP* | Unknown |
| 01572 |  | Outer membrane efflux protein |
| 01573 |  | ABC transporter |
| 01574 |  | Membrane efflux protein |
| 01575 | *fpvI* | ECF sigma factor required for expression of *fpvA* |
| 01576 | *pvdA* | L-ornithine hydroxylase |
| 02454 | *pvdQ* | Acylase |
