# Supplementary material for: The Complete Genome Sequence of the Plant Growth-Promoting Bacterium Pseudomonas sp. UW4
Source: PLoS One. 2013 Mar 13;8(3):e58640. doi: 10.1371/journal.pone.0058640 (PMC3596284; doi:10.1371/journal.pone.0058640)
Supplement: Table S7 — P. sp. UW4 Multidrug Efflux Systems. (DOCX) [file pone.0058640.s010.docx]

Table S7. *Pseudomonas* sp*.* UW4 Multidrug Efflux Systems.

| PputUW4_ | Product |
| --- | --- |
| 00123 | multidrug efflux MFS transporter |
| 00124 | multidrug efflux MFS membrane fusion protein |
| 00125 | multidrug efflux MFS outer membrane protein |
| 00133 | fusaric acid resistance protein |
| 00135 | multidrug resistance efflux pump protein |
| 00136 | multidrug efflux outer membrane protein |
| 00178 | multidrug RND transporter, membrane fusion protein |
| 00179 | multidrug RND transporter, membrane fusion protein |
| 00180 | acriflavin resistance protein |
| 00501 | small multidrug resistance protein SugE |
| 01066 | multidrug RND transporter, membrane fusion protein |
| 01067 | acriflavin resistance protein |
| 01211 | multidrug efflux RND outer membrane protein |
| 01212 | multidrug efflux RND inner membrane transporter |
| 01213 | multidrug efflux RND membrane fusion protein |
| 01591 | EmrB/QacA family drug resistance transporter |
| 01592 | multidrug resistance protein (HlyD family), EmrA-like secretion |
| 01894 | multidrug resistance efflux protein |
| 01895 | EmrB/QacA subfamily drug resistance transporter |
| 02110 | multidrug efflux system outer membrane protein |
| 02111 | RND family efflux transporter MFP subunit |
| 02112 | acriflavin resistance protein |
| 02139 | acriflavin resistance protein |
| 02140 | RND family efflux transporter MFP subunit |
| 02141 | RND efflux system outer membrane lipoprotein |
| 02438 | multidrug efflux system transmembrane protein |
| 02439 | multidrug efflux RND membrane fusion protein |
| 02531 | fusaric acid resistance protein |
| 02533 | multidrug resistance efflux pump protein |
| 02534 | multidrug efflux system outer membrane protein |
| 02535 | multidrug efflux RND transporter permease MexF |
| 02536 | RND family efflux transporter MFP subunit |
| 02810 | multidrug efflux system outer membrane protein |
| 02811 | multidrug efflux system transmembrane protein |
| 02812 | multidrug efflux system transmembrane protein |
| 02813 | multidrug efflux system inner membrane protein |
| 02949 | ABC efflux system outer membrane protein |
| 02950 | ABC efflux system ATP-binding protein |
| 02951 | ABC efflux system permease |
| 03466 | multidrug resistance efflux pump |
| 03467 | EmrB/QacA family drug resistance transporter |
| 03528 | multidrug ABC transporter ATPase/permease |
| 03531 | multidrug efflux transporter |
| 03751 | small multidrug resistance protein |
| 03983 | multidrug ABC transporter ATP-binding protein/permease |
| 04056 | ABC-type multidrug transport system, permease component |
| 04057 | ABC-type multidrug transport system, ATPase component |
| 04378 | RND family multidrug transporter membrane fusion protein |
| 04379 | RND multidrug efflux transporter |
| 04486 | fusaric acid resistance protein |
| 04488 | fusaric acid resistance protein |
| 04489 | fusaric acid resistance protein FusA |
| 04491 | RND family multidrug transporter membrane fusion protein |
| 04492 | multidrug efflux system transmembrane protein |
| 05151 | multidrug resistance transporter, Bcr/CflA family |
| 05301 | multidrug resistance-transport membrane protein |
| 05419 | permease |
| 05420 | multidrug efflux system outer membrane protein |
| 05421 | multidrug resistance efflux pump |
| 05422 | membrane protein |
