# Supplementary material for: The Complete Genome Sequence of the Plant Growth-Promoting Bacterium Pseudomonas sp. UW4
Source: PLoS One. 2013 Mar 13;8(3):e58640. doi: 10.1371/journal.pone.0058640 (PMC3596284; doi:10.1371/journal.pone.0058640)
Supplement: Table S8 — Genes potentially involved in metal resistance of P. sp, UW4. (DOCX) [file pone.0058640.s011.docx]

Table S8. Genes potentially involved in metal resistance of *Pseudomonas* sp*.* UW4.

| Metal | Gene | PputUW4_ | Product |
| --- | --- | --- | --- |
| Nickel | *nikA3* | 00746 | nickel ABC transporter periplasmic nickel-binding protein |
|  | *nikA2* | 00745 | nickel ABC transporter periplasmic nickel-binding protein |
|  | *nikA1* | 00743 | nickel ABC transporter periplasmic nickel-binding protein |
|  | *nikB* | 00742 | nickel transporter permease |
|  | *nikC* | 00741 | nickel transporter permease |
|  | *nikD* | 00740 | nickel import ATP-binding protein |
|  | *nikE* | 00739 | nickel import ATP-binding protein |
| Copper | *copP* | 00581 | heavy metal transport/detoxification protein |
|  | *cueA* | 00579 | copper-translocating P-type ATPase |
|  | *cueR* | 00578 | MerR family transcriptional regulator |
|  | *copA* | 03484 | copper resistance protein |
|  | *copB* | 03485 | copper resistance protein |
|  | *copC* | 03486 | copper resistance protein |
|  | *copD* | 03487 | copper resistance protein |
|  | *cinQ* | 03498 | 7-cyano-7-deazaguanine (pre-Q0) reductase |
|  | *cinA* | 03499 | copper-containing azurin-like protein |
|  | *cinR* | 03500 | two-component heavy metal response transcriptional regulator |
|  | *cinS* | 03501 | heavy metal sensor histidine kinase |
|  |  | 02449 | involved in survival in the presence of high bioavailable Cu(II) |
|  |  | 02046 | heavy metal sensor signal transduction histidine kinase |
|  |  | 02047 | two-component heavy metal response transcriptional regulator |
|  |  | 04493 | two-component heavy metal response transcriptional regulator |
|  |  | 04494 | heavy metal sensor signal transduction histidine kinase |
| Cadmium | *cadA1* | 05166 | cadmium translocating P-type ATPase |
|  | *cadR* | 05167 | MerR family transcriptional regulator |
|  | *cadA2* | 05407 | cadmium-translocating P-type ATPase |
| Zinc |  | 01616 | metallothionein |
|  | *znuA* | 00067 | zinc ABC transporter periplasmic protein |
|  | *znuB* | 00064 | zinc ABC transporter permease |
|  | *znuC* | 00065 | zinc import ATP-binding protein |
|  | *zur* | 00066 | ferric uptake regulator family protein |
| Molybdate | *modA* | 02399 | molybdate-binding periplasmic protein |
|  | *modB* | 02398 | molybdate ABC transporter permease |
|  | *modC* | 02397 | molybdate ABC transporter ATP-binding protein |
|  |  | 04985 | ModE family transcriptonal regulator |
| Cobalt | *cbtA* | 02359 | cobalt transporter subunit A |
|  | *cbtB* | 02360 | cobalt transporter subunit B |
| Arsenate | *arsR* | 02251 | arsenical resistance operon repressor |
|  | *arsB* | 02250 | arsenite efflux transporter |
|  | *arsC1* | 02249 | arsenate reductase |
|  | *arsH* | 02248 | NADPH-dependent FMN reductase |
|  | *arsC2* | 01082 | arsenate reductase |
|  | *arsC3* | 04117 | arsenate reductase |
| Chromate | *chrA* | 03067 | chromate transporter |
|  | oscA | 00153 | hypothetical protein |
