# Supplementary material for: The Complete Genome Sequence of the Plant Growth-Promoting Bacterium Pseudomonas sp. UW4
Source: PLoS One. 2013 Mar 13;8(3):e58640. doi: 10.1371/journal.pone.0058640 (PMC3596284; doi:10.1371/journal.pone.0058640)
Supplement: Table S9 — Protein secretion systems in P. sp, UW4. (DOCX) [file pone.0058640.s012.docx]

Table S9. Protein secretion systems in *Pseudomonas* sp*.* UW4.

| System | Gene | PputUW4_ |
| --- | --- | --- |
| *(a) Sec secretory pathway* |  |  |
| Chaperone | *secB* | 00279 |
| ATPase | *secA* | 04316 |
| TM complex | *secY* | 04864 |
|  | *secE* | 04897 |
|  | *secG* | 00703 |
| Auxiliary proteins | *secD* | 00920 |
|  | *secF* | 00921 |
|  | *yajC* | 00919 |
| Membrane insertion proteins | *ftsY* | 05135 |
|  | *ffh* | 04434 |
|  | *yidC* | 05440 |
| Spase I | *lepB* | 00957 |
| Spase II | *lspA* | 04667 |
| *(b) Twin arginine targeting (Tat) secretory pathway* |  |  |
|  | *tatA* | 00321 |
|  | *tatB* | 00320 |
|  | *tatC* | 00319 |
| *(c) Large conductance mechanosensitive ion channel* |  |  |
| Channel | *mscL* | 04705 |
| *(d) Type I secretory system* |  |  |
| TolC family outer membrane protein |  | 00115 |
|  |  | 01719 |
|  |  | 03951 |
| HylD family membrane fusion protein |  | 00117 |
|  |  | 01720 |
|  |  | 02632 |
|  |  | 03953 |
| ATPase |  | 00116 |
|  |  | 01721 |
|  |  | 02633 |
|  |  | 03952 |
| Putative substrate: RTX toxins and related Ca^2+^ binding proteins |  | 00114 |
|  |  | 01722 |
|  |  | 02631 |
|  |  | 03950 |
| *(e) Type II secretory system* |  |  |
| Secretin | *gspD* | 03285 |
| Inner membrane protein | *gspF* | 03298 |
|  | *gspG* | 03282 |
|  |  | 03283 |
|  |  | 03284 |
|  |  | 03297 |
| APTase | *gspE* | 03290 |
|  |  | 05224 |
| *(f) Type III secretory system* |  |  |
| HopJ type III effector protein |  | 00807 |
| Apparatus protein | *orgAB* | 03613 |
| Apparatus lipoprotein | *prgK* | 03614 |
| Needle complex protein | *prgJ* | 03615 |
| Cytoplasmic protein | *prgI* | 03616 |
| Needle complex protein | *prgH* | 03617 |
| Cell invasion protein | *iagB* | 03618 |
| Invasion protein regulator | *hilA* | 03619 |
| Acyl carrier protein | *iacP* | 03620 |
| Cell invasion protein | *sipD* | 03621 |
| Invasin | *sipC* | 03622 |
|  | *sipB* | 03623 |
| Chaperone | *sicA* | 03624 |
| Surface presentation of antigens protein | *spaS* | 03625 |
|  | *spaR* | 03626 |
|  | *spaQ* | 03627 |
|  | *spaP* | 03628 |
|  | *spaO* | 03629 |
| Invasion protein | *invJ* | 03630 |
| Secretory apparatus | *invI* | 03631 |
| Secretory apparatus ATP synthase | *invC* | 03632 |
| Invasion protein | *invB* | 03633 |
|  | *invA* | 03634 |
|  | *invE* | 03635 |
|  | *invG* | 03636 |
|  | *invF* | 03637 |
| *(g) Type V secretory system* |  |  |
| Autotransporters | *estA* | 04920 |
|  |  | 02797 |
|  |  | 00217 |
| Two-partner secretion | *lepA* | 00956 |
|  | *lepB* | 00957 |
| *(h) Type VI secretory system* |  |  |
| OmpA/MotB domain-containing protein |  | 03071 |
| Lysozyme-like protein |  | 03072 |
| Lipoprotein |  | 03073 |
| Secretion protein |  | 03074 |
|  |  | 03075 |
| Secretion-associated protein |  | 03076 |
| ImcF domain-containing protein |  | 03077 |
| Hypothetical protein |  | 03078 |
| PAAR repeat-containing protein |  | 03079 |
| Hypothetical protein |  | 03080 |
|  |  | 03081 |
|  |  | 03082 |
| Vgr family protein |  | 03083 |
| ClpV1 family type VI secretion ATPase |  | 03084 |
| Hcp1 family effector |  | 03085 |
| OmpA/MotB domain-containing protein |  | 03086 |
| Hypothetical protein |  | 03087 |
| Secretion protein |  | 03088 |
| EvpB family secretion protein |  | 03089 |
| Secretion protein |  | 03090 |
| Vgr family protein |  | 03279 |
