# Supplementary material for: The Complete Genome Sequence of the Plant Growth-Promoting Bacterium Pseudomonas sp. UW4
Source: PLoS One. 2013 Mar 13;8(3):e58640. doi: 10.1371/journal.pone.0058640 (PMC3596284; doi:10.1371/journal.pone.0058640)
Supplement: Table S10 — Type IV Pilus Genes in P. sp, UW4. (DOCX) [file pone.0058640.s013.docx]

Table S10. Type IV Pilus Genes in *Pseudomonas* sp*.* UW4

| Gene | Gene Product | PputUW4_ |
| --- | --- | --- |
| *pilM* | type IV pili biogenesis protein PilM | 00344 |
| *pilN* | type IV pili biogenesis protein PilN | 00345 |
| *pilO* | type IV pili biogenesis protein PilO | 00346 |
| *pilP* | type IV pili biogenesis protein PilP | 00347 |
| *pilQ* | type IV pili biogenesis protein PilQ | 00348 |
| *pilF* | type IV pili biogenesis protein PilF | 00937 |
| *pilZ* | type IV pilus assembly PilZ | 03505 |
| *pilZ* | type IV pilus assembly PilZ | 04334 |
| *fimD* | fimbrial biogenesis outer membrane usher protein | 04449 |
| *pilA* | type IV pili prepilin PilA | 04640 |
| *pilC* | type IV fimbrial assembly protein | 04641 |
| *pilD* | type IV pili prepilin peptidase PilD/XcpA | 04642 |
| *pilE* | type IV pili biogenesis protein PilE | 04658 |
| *pilX* | type IV pilus assembly protein | 04660 |
| *pilW* | type IV pilus assembly protein | 04661 |
| *pilV* | type IV fimbrial biogenesis protein PilV | 04662 |
| *fimT* | type IV pilin | 04663 |
| *fimT* | type IV pili biogenesis protein | 04664 |
| *traX* | conjugal transfer pilus acetylation protein | 05013 |
| *pilL/chpA* | type IV pili sensor histidine kinase/response regulator | 05101 |
| *pilJ* | type IV pili methyl-accepting chemotaxis transducer | 05102 |
| *pilI* | type IV pili signal transduction protein PilI | 05103 |
| *pilH* | type IV pili response regulator PilH | 05104 |
| *pilG* | type IV pili response regulator PilG | 05105 |
