# Supplementary material for: The Complete Genome Sequence of the Plant Growth-Promoting Bacterium Pseudomonas sp. UW4
Source: PLoS One. 2013 Mar 13;8(3):e58640. doi: 10.1371/journal.pone.0058640 (PMC3596284; doi:10.1371/journal.pone.0058640)
Supplement: Table S11 — Putative Orthologous Relations Between UW4 and completely sequenced Pseudomonas genomes. (DOCX) [file pone.0058640.s014.docx]

Table S11. Putative Orthologous Relations Between UW4 and Completely Sequenced *Pseudomonas* Genomes

| Species | Putative orthologous shared with *P.* sp. UW4, no. (%) |
| --- | --- |
| *P. fluorescens* Pf0-1 | 4113 (76) |
| *P. protegens* Pf-5 | 3789 (70) |
| *P. fluorescens* SBW25 | 3595 (66) |
| *P. putida* KT2440 | 3356 (62) |
| *P. putida* GB-1 | 3434 (63) |
| *P. putida* F1 | 3435 (63) |
| *P. putida* BIRD-1 | 3394 (62) |
| *P. putida* W619 | 3405 (63) |
| *P. putida* S16 | 3466 (64) |
| *P. brassicacearum* NFM-421 | 3815 (69) |
| *P. entomophila* L48 | 3387 (62) |
| *P. syringae* DC3000 | 3015 (55) |
| *P. syringae* 1448a | 2992 (55) |
| *P. syringae* B728a | 3061 (56) |
| *P. mendocina* NK-01 | 2918 (54) |
| *P. stutzeri* A1501 | 2414 (44) |
| *P. aeruginosa* LESB58 | 3175 (58) |
| *P. aeruginosa* PAO1 | 3182 (58) |
| *P. aeruginosa* UCBPP-PA14 | 3185 (58) |
| *P. aeruginosa* PA7 | 3182 (58) |
