# Supplementary material for: The Complete Genome Sequence of the Plant Growth-Promoting Bacterium Pseudomonas sp. UW4
Source: PLoS One. 2013 Mar 13;8(3):e58640. doi: 10.1371/journal.pone.0058640 (PMC3596284; doi:10.1371/journal.pone.0058640)
Supplement: Table S12 — Predicted CDSs that share similarities with other Pseudomonas sp. (DOCX) [file pone.0058640.s015.docx]

Table S12. Predicted CDSs that share similarities with other *Pseudomonas* spp.

| PputUW4_ | product |
| --- | --- |
| 00241 | transcription repressor protein |
| 00799 | hypothetical protein |
| 00915 | type III restriction protein res subunit |
| 01232 | hypothetical protein |
| 01652 | short chain dehydrogenase |
| 01653 | dehydratase |
| 01655 | 3-ketosteroid-5-isomerase |
| 01659 | 2,6-dioxo-6-phenylhexa-3-enoate hydrolase |
| 01667 | short-chain dehydrogenase/reductase SDR |
| 01668 | lipase |
| 01673 | hypothetical protein |
| 01674 | 3-oxosteroid-delta-1-dehydrogenase |
| 01675 | short chain dehydrogenase |
| 01687 | 2,3-dihydroxybiphenyl-1,2-dioxygenase |
| 01688 | CoA-transferase subunit alpha |
| 01689 | CoA-transferase, beta subunit |
| 01690 | enoyl-CoA hydratase |
| 01691 | 2-nitropropane dioxygenase |
| 01692 | enoyl-CoA hydratase |
| 01693 | acyl-CoA dehydrogenase |
| 01694 | acyl-CoA dehydrogenase |
| 01696 | short chain dehydrogenase |
| 01737 | LysR family transcriptional regulator |
| 01812 | leucyl-tRNA synthetase |
| 01890 | glutamine synthetase |
| 01989 | AraC family transcriptional regulator |
| 02071 | AMP-dependent synthetase and ligase |
| 02073 | AMP-dependent synthetase and ligase |
| 02076 | acyl-CoA dehydrogenase |
| 02078 | enoyl-CoA hydratase |
| 02192 | diguanylate cyclase |
| 02252 | ribosomal-protein-alanine acetyltransferase |
| 02264 | exported protein |
| 02265 | alpha/beta fold family hydrolase |
| 02275 | phenylserine aldolase |
| 02303 | long-chain-fatty-acid--CoA ligase |
| 02563 | plasmid stabilization system protein |
| 02605 | phage integrase |
| 02617 | AraC family transcriptional regulator |
| 02619 | alpha/beta hydrolase fold family protein |
| 02641 | hypothetical protein |
| 03042 | betaine aldehyde dehydrogenase |
| 03139 | aldehyde dehydrogenase |
| 03252 | hypothetical protein |
| 03274 | MutT/nudix-family hydrolase |
| 03549 | dTDP-4-dehydrorhamnose 3,5-epimerase |
| 03588 | hypothetical protein |
| 03612 | hypothetical protein |
| 03613 | oxygen-regulated gene required for bacterial internalization |
| 03614 | type III secretion apparatus lipoprotein |
| 03615 | type III secretion system needle complex protein |
| 03616 | type III secretion cytoplasmic protein |
| 03617 | type III secretion system needle complex protein |
| 03619 | invasion protein regulator |
| 03620 | acyl carrier protein |
| 03621 | type III effector protein cell invasion protein |
| 03622 | invasin |
| 03623 | Invasin |
| 03624 | chaperone protein SicA |
| 03625 | surface presentation of antigens protein |
| 03626 | surface presentation of antigens protein |
| 03627 | surface presentation of antigens protein |
| 03629 | hypothetical protein |
| 03630 | hypothetical protein |
| 03631 | hypothetical protein |
| 03635 | invasion protein |
| 03637 | invasion protein |
| 03916 | hypothetical protein |
| 04957 | transcriptional regulator |
| 04958 | hypothetical protein |
| 05040 | hypothetical protein |
