# Supplementary material for: The Complete Genome Sequence of the Plant Growth-Promoting Bacterium Pseudomonas sp. UW4
Source: PLoS One. 2013 Mar 13;8(3):e58640. doi: 10.1371/journal.pone.0058640 (PMC3596284; doi:10.1371/journal.pone.0058640)
Supplement: Table S13 — Putative unique CDSs in P. sp. UW4. (DOCX) [file pone.0058640.s016.docx]

Table S13. Putative unique CDSs in *Pseudomonas* sp*.* UW4

| PputUW4_ | product |
| --- | --- |
| 00044 | hypothetical protein |
| 00101 | hypothetical protein |
| 00141 | hypothetical protein |
| 00142 | hypothetical protein |
| 00143 | hypothetical protein |
| 00144 | peptidase |
| 00149 | IS3 family transposase |
| 00202 | hypothetical protein |
| 00217 | Extracellular serine protease |
| 00229 | hypothetical protein |
| 00237 | hypothetical protein |
| 00357 | hypothetical protein |
| 00486 | hypothetical protein |
| 00497 | hypothetical protein |
| 00498 | hypothetical protein |
| 00537 | hypothetical protein |
| 00542 | hypothetical protein |
| 00543 | hypothetical protein |
| 00571 | hypothetical protein |
| 00592 | hypothetical protein |
| 00633 | hypothetical protein |
| 00637 | hypothetical protein |
| 00642 | hypothetical protein |
| 00643 | hypothetical protein |
| 00644 | hypothetical protein |
| 00648 | hypothetical protein |
| 00655 | hypothetical protein |
| 00674 | hypothetical protein |
| 00723 | hypothetical protein |
| 00724 | hypothetical protein |
| 00734 | hypothetical protein |
| 00737 | hypothetical protein |
| 00738 | hypothetical protein |
| 00858 | hypothetical protein |
| 00863 | hypothetical protein |
| 00891 | hypothetical protein |
| 00892 | hypothetical protein |
| 00900 | hypothetical protein |
| 00909 | ion channel family protein |
| 00923 | hypothetical protein |
| 00965 | hypothetical protein |
| 01011 | hypothetical protein |
| 01025 | hypothetical protein |
| 01027 | hypothetical protein |
| 01068 | hypothetical protein |
| 01180 | hypothetical protein |
| 01196 | hypothetical protein |
| 01210 | hypothetical protein |
| 01215 | hypothetical protein |
| 01231 | LuxR family transcriptional regulator |
| 01233 | hypothetical protein |
| 01235 | hypothetical protein |
| 01263 | hypothetical protein |
| 01264 | hypothetical protein |
| 01311 | hypothetical protein |
| 01372 | hypothetical protein |
| 01390 | K+-transporting ATPase subunit F |
| 01411 | hypothetical protein |
| 01485 | hypothetical protein |
| 01486 | hypothetical protein |
| 01487 | hypothetical protein |
| 01496 | hypothetical protein |
| 01544 | hypothetical protein |
| 01553 | hypothetical protein |
| 01554 | hypothetical protein |
| 01556 | hypothetical protein |
| 01564 | hypothetical protein |
| 01616 | metallothionein |
| 01663 | hypothetical protein |
| 01684 | hypothetical protein |
| 01703 | hypothetical protein |
| 01716 | hypothetical protein |
| 01723 | hypothetical protein |
| 01727 | hypothetical protein |
| 01740 | hypothetical protein |
| 01758 | hypothetical protein |
| 01809 | Class II Aldolase/Adducin Family Protein |
| 01874 | hypothetical protein |
| 01918 | hypothetical protein |
| 01929 | hypothetical protein |
| 01943 | hypothetical protein |
| 01955 | hypothetical protein |
| 01992 | hypothetical protein |
| 02005 | hypothetical protein |
| 02011 | IS3 family transposase |
| 02015 | hypothetical protein |
| 02017 | hypothetical protein |
| 02042 | aldehyde dehydrogenase family protein |
| 02043 | hypothetical protein |
| 02093 | hypothetical protein |
| 02094 | hypothetical protein |
| 02096 | hypothetical protein |
| 02102 | hypothetical protein |
| 02120 | IS1182 family transposase |
| 02121 | ThiF family protein |
| 02122 | hypothetical protein |
| 02124 | hypothetical protein |
| 02125 | hypothetical protein |
| 02126 | hypothetical protein |
| 02127 | hypothetical protein |
| 02130 | hypothetical protein |
| 02131 | IS1182 family transposase |
| 02142 | hypothetical protein |
| 02159 | IS1182 family transposase |
| 02162 | hypothetical protein |
| 02164 | hypothetical protein |
| 02166 | hypothetical protein |
| 02174 | hypothetical protein |
| 02178 | hypothetical protein |
| 02182 | heme peroxidase |
| 02183 | hypothetical protein |
| 02187 | hypothetical protein |
| 02197 | hypothetical protein |
| 02198 | hypothetical protein |
| 02200 | hypothetical protein |
| 02211 | LysR family transcriptional regulator |
| 02212 | hypothetical protein |
| 02218 | IS1182 family transposase |
| 02279 | hemolysin-type calcium-binding region |
| 02280 | hypothetical protein |
| 02283 | hypothetical protein |
| 02302 | LuxR family ATP-dependent transcriptional regulator |
| 02348 | hypothetical protein |
| 02351 | hypothetical protein |
| 02366 | hypothetical protein |
| 02390 | hypothetical protein |
| 02392 | hypothetical protein |
| 02410 | hypothetical protein |
| 02442 | hypothetical protein |
| 02452 | hypothetical protein |
| 02476 | hypothetical protein |
| 02515 | hypothetical protein |
| 02529 | hypothetical protein |
| 02530 | hypothetical protein |
| 02532 | hypothetical protein |
| 02565 | hypothetical protein |
| 02569 | hypothetical protein |
| 02583 | hypothetical protein |
| 02584 | AraC family transcriptional regulator |
| 02596 | hypothetical protein |
| 02602 | AraC family transcriptional regulator |
| 02603 | hypothetical protein |
| 02604 | hypothetical protein |
| 02606 | hypothetical protein |
| 02615 | hypothetical protein |
| 02616 | hypothetical protein |
| 02627 | hypothetical protein |
| 02628 | hypothetical protein |
| 02652 | hypothetical protein |
| 02653 | hypothetical protein |
| 02663 | hypothetical protein |
| 02669 | ion transport 2 domain-containing protein |
| 02683 | hypothetical protein |
| 02700 | hypothetical protein |
| 02720 | hypothetical protein |
| 02722 | hypothetical protein |
| 02723 | CadC family transcriptional regulator |
| 02724 | hypothetical protein |
| 02725 | hypothetical protein |
| 02726 | AraC family transcriptional regulator |
| 02727 | hypothetical protein |
| 02728 | OmpA-like transmembrane domain protein |
| 02729 | hypothetical protein |
| 02745 | hypothetical protein |
| 02756 | DNA-binding response regulator |
| 02757 | hypothetical protein |
| 02772 | hypothetical protein |
| 02802 | hypothetical protein |
| 02840 | hypothetical protein |
| 02865 | hypothetical protein |
| 02923 | hypothetical protein |
| 02988 | hypothetical protein |
| 02989 | hypothetical protein |
| 02996 | IS110 family transposase |
| 03022 | hypothetical protein |
| 03037 | hypothetical protein |
| 03043 | hypothetical protein |
| 03059 | hypothetical protein |
| 03080 | hypothetical protein |
| 03083 | type VI secretion system Vgr family protein |
| 03093 | hypothetical protein |
| 03098 | hypothetical protein |
| 03125 | hypothetical protein |
| 03128 | hypothetical protein |
| 03136 | hypothetical protein |
| 03137 | hypothetical protein |
| 03140 | hypothetical protein |
| 03141 | hypothetical protein |
| 03154 | hypothetical protein |
| 03184 | hypothetical protein |
| 03186 | hypothetical protein |
| 03209 | glutamine amidotransferase class-I |
| 03221 | hypothetical protein |
| 03241 | hypothetical protein |
| 03250 | hypothetical protein |
| 03265 | hypothetical protein |
| 03275 | hypothetical protein |
| 03281 | hypothetical protein |
| 03292 | hypothetical protein |
| 03299 | hypothetical protein |
| 03306 | hypothetical protein |
| 03307 | hypothetical protein |
| 03319 | hypothetical protein |
| 03324 | hypothetical protein |
| 03344 | hypothetical protein |
| 03354 | hypothetical protein |
| 03411 | hypothetical protein |
| 03412 | hypothetical protein |
| 03413 | hypothetical protein |
| 03416 | hypothetical protein |
| 03473 | hypothetical protein |
| 03477 | hypothetical protein |
| 03481 | hypothetical protein |
| 03542 | hypothetical protein |
| 03543 | hypothetical protein |
| 03555 | hypothetical protein |
| 03582 | hypothetical protein |
| 03583 | hypothetical protein |
| 03585 | hypothetical protein |
| 03586 | hypothetical protein |
| 03589 | hypothetical protein |
| 03590 | hypothetical protein |
| 03592 | hypothetical protein |
| 03611 | hypothetical protein |
| 03672 | hypothetical protein |
| 03673 | hypothetical protein |
| 03674 | surface antigen protein |
| 03675 | surface antigen protein |
| 03676 | hypothetical protein |
| 03678 | hypothetical protein |
| 03758 | hypothetical protein |
| 03763 | hypothetical protein |
| 03807 | hypothetical protein |
| 03949 | hypothetical protein |
| 03962 | hypothetical protein |
| 03963 | hypothetical protein |
| 03991 | aldehyde dehydrogenase family protein |
| 04017 | hypothetical protein |
| 04064 | hypothetical protein |
| 04078 | hypothetical protein |
| 04108 | hypothetical protein |
| 04109 | hypothetical protein |
| 04149 | hypothetical protein |
| 04152 | hypothetical protein |
| 04158 | hypothetical protein |
| 04159 | hypothetical protein |
| 04161 | hypothetical protein |
| 04167 | hypothetical protein |
| 04189 | hypothetical protein |
| 04227 | hypothetical protein |
| 04229 | hypothetical protein |
| 04306 | hypothetical protein |
| 04331 | hypothetical protein |
| 04357 | hypothetical protein |
| 04358 | hypothetical protein |
| 04495 | hypothetical protein |
| 04513 | hypothetical protein |
| 04514 | hypothetical protein |
| 04620 | hypothetical protein |
| 04691 | hypothetical protein |
| 04693 | hypothetical protein |
| 04823 | hypothetical protein |
| 04906 | hypothetical protein |
| 04959 | hypothetical protein |
| 05015 | hypothetical protein |
| 05016 | hypothetical protein |
| 05020 | hypothetical protein |
| 05084 | hypothetical protein |
| 05206 | hypothetical protein |
| 05214 | hypothetical protein |
| 05346 | hypothetical protein |
