# Supplementary material for: The Complete Genome Sequence of the Plant Growth-Promoting Bacterium Pseudomonas sp. UW4
Source: PLoS One. 2013 Mar 13;8(3):e58640. doi: 10.1371/journal.pone.0058640 (PMC3596284; doi:10.1371/journal.pone.0058640)
Supplement: Table S14 — Predicted UW4 CDSs that share sequence similarities to those in other genera only. (DOCX) [file pone.0058640.s017.docx]

Table S14. Predicted UW4 CDSs that share sequence similarities to those in other genera only

| PputUW4_ | product |
| --- | --- |
| 00442 | hypothetical protein |
| 00523 | hypothetical protein |
| 00641 | hypothetical protein |
| 00654 | ADP-Ribosylglycohydrolase |
| 00663 | hypothetical protein |
| 00736 | hypothetical protein |
| 00860 | hypothetical protein |
| 00861 | hypothetical protein |
| 00865 | radical SAM family protein |
| 00866 | branched-chain amino acid aminotransferase |
| 00867 | hypothetical protein |
| 00868 | hypothetical protein |
| 00869 | hypothetical protein |
| 00870 | GntR family transcriptional regulator with aminotransferase domain |
| 00881 | histidine kinase |
| 00882 | two component transcriptional regulator |
| 00883 | methionine-R-sulfoxide reductase |
| 00884 | methionine sulfoxide reductase A |
| 00889 | hypothetical protein |
| 01330 | Glucose/sorbosone dehydrogenases |
| 01331 | tat (twin-arginine translocation) pathway signalsequence domain protein |
| 01370 | hypothetical protein |
| 01371 | hypothetical protein |
| 01373 | hypothetical protein |
| 01558 | hypothetical protein |
| 01590 | hypothetical protein |
| 01650 | acyl-CoA dehydrogenase |
| 01651 | acyl-CoA dehydrogenase |
| 01656 | hypothetical protein |
| 01657 | hypothetical protein |
| 01658 | oxidoreductase, short chain dehydrogenase/reductase family |
| 01665 | LuxR family transcriptional regulator |
| 01666 | rieske [2Fe-2S] domain protein |
| 01671 | 5'-nucleotidase SurE |
| 01676 | acyl-CoA synthetase |
| 01677 | hypothetical protein |
| 01678 | FAD-binding oxidoreductase |
| 01680 | fumarate reductase/succinate dehydrogenase |
| 01682 | acyl-CoA dehydrogenase |
| 01683 | flavin-dependent oxidoreductase |
| 01726 | 17 kDa surface antigen |
| 01732 | hypothetical protein |
| 01733 | arylsulfatase |
| 01734 | multiple antibiotic resistance (MarC)-like protein |
| 01738 | AMP-dependent synthetase and ligase |
| 01741 | enoyl-CoA hydratase |
| 01805 | Citrate lyase subunit beta |
| 01806 | MaoC Domain Protein Dehydratase |
| 01807 | ornithine cyclodeaminase |
| 01808 | D-3-phosphoglycerate dehydrogenase |
| 01810 | histidinol-phosphate aminotransferase |
| 01855 | LuxR family transcriptional regulator |
| 01856 | amidohydrolase family protein |
| 01860 | hypothetical protein |
| 01861 | D-alanyl-D-alanine dipeptidase |
| 01880 | hypothetical protein |
| 01887 | amino acid permease family protein |
| 02008 | AraC family transcriptional regulator |
| 02018 | hypothetical protein |
| 02026 | hypothetical protein |
| 02029 | Arginine/lysine/ornithine decarboxylases |
| 02030 | putrescine transporter |
| 02049 | hypothetical protein |
| 02050 | hypothetical protein |
| 02057 | dihydroxy-acid dehydratase |
| 02062 | TetR family transcriptional regulator |
| 02063 | short-chain dehydrogenase/reductase SDR |
| 02064 | MaoC-like dehydratase |
| 02065 | hypothetical protein |
| 02066 | thiolase |
| 02068 | LuxR family MalT-like ATP-dependent transcriptional regulator |
| 02070 | enoyl-CoA hydratase/carnithine racemase |
| 02072 | thioesterase superfamily protein |
| 02075 | thiolase |
| 02077 | acyl-CoA dehydrogenase |
| 02083 | alcohol dehydrogenase class III |
| 02086 | hypothetical protein |
| 02087 | hypothetical protein |
| 02088 | AraC family transcriptional regulator |
| 02092 | hypothetical protein |
| 02116 | RND family efflux transporter, MFP subunit |
| 02118 | peptide ABC transporter permease |
| 02119 | peptide ABC transporter permease |
| 02129 | hypothetical protein |
| 02135 | HxlR family transcriptional regulator |
| 02137 | glutathione-dependent formaldehyde-activating GFA |
| 02138 | hypothetical protein |
| 02140 | RND family efflux transporter MFP subunit |
| 02141 | RND efflux system outer membrane lipoprotein |
| 02143 | transcriptional regulator ATPase, winged helix family |
| 02145 | TetR family transcriptional regulator |
| 02147 | TetR family transcriptional regulator |
| 02149 | fumarylacetoacetate (FAA) hydrolase |
| 02152 | hypothetical protein |
| 02169 | hypothetical protein |
| 02180 | LuxR family transcriptional regulator |
| 02181 | histidine kinase family protein |
| 02184 | sensor histidine kinase |
| 02185 | sensor histidine kinase |
| 02186 | two component LuxR family transcriptional regulator |
| 02190 | hypothetical protein |
| 02201 | hypothetical protein |
| 02234 | hypothetical protein |
| 02255 | hypothetical protein |
| 02259 | hypothetical protein |
| 02278 | twin-arginine translocation pathway signal |
| 02281 | patatin-like phospholipase family protein |
| 02293 | thiolase |
| 02294 | MaoC-like dehydratase |
| 02295 | MaoC-like dehydratase |
| 02296 | 3-oxoacyl-[acyl-carrier-protein] reductase |
| 02326 | major facilitator superfamily MFS_1 |
| 02549 | hypothetical protein |
| 02566 | hypothetical protein |
| 02568 | hypothetical protein |
| 02580 | hypothetical protein |
| 02581 | hypothetical protein |
| 02582 | hypothetical protein |
| 02586 | LuxR family ATP-dependent transcriptional regulator |
| 02587 | acetyl-CoA acetyltransferase |
| 02592 | Radical SAM domain protein |
| 02593 | oxidoreductase |
| 02594 | arylsulfatase |
| 02595 | 2,4-dihydroxyacetophenone dioxygenase |
| 02607 | hypothetical protein |
| 02620 | major facilitator superfamily protein |
| 02621 | enoyl-CoA hydratase/isomerase |
| 02622 | AMP-dependent synthetase and ligase |
| 02623 | carbon monoxide dehydrogenase small subunit |
| 02625 | carbon-monoxide dehydrogenase, large subunit |
| 02671 | TetR family transcriptional regulator |
| 02672 | hypothetical protein |
| 02676 | glutathione-dependent formaldehyde-activating GFA |
| 02687 | hypothetical protein |
| 02688 | major facilitator family protein |
| 02696 | hypothetical protein |
| 02697 | MarR family transcriptional regulator |
| 02703 | isochorismatase family protein |
| 02708 | cytochrome b561 family protein |
| 02709 | hypothetical protein |
| 02711 | hypothetical protein |
| 02712 | cytochrome c oxidase subunit III |
| 02713 | cytochrome c oxidase subunit III |
| 02714 | cytochrome c oxidase, subunit I |
| 02715 | cytochrome c oxidase subunit II |
| 02718 | hypothetical protein |
| 02719 | hypothetical protein |
| 02740 | ABC transporter permease protein |
| 02741 | ABC transporter permease protein |
| 02742 | ABC transporter family protein |
| 02743 | ABC transporter family protein |
| 02744 | ABC transporter substrate-binding protein |
| 02842 | hypothetical protein |
| 02990 | LuxR family two component transcriptional regulator |
| 03007 | IclR family transcriptional regulator |
| 03019 | nitrilase |
| 03021 | glutathione S-transferase |
| 03038 | spermidine/putrescine ABC transporter ATPase subunit |
| 03039 | spermidine/putrescine-binding periplasmic protein |
| 03053 | prolyl-tRNA synthetase |
| 03081 | hypothetical protein |
| 03092 | sulfate transporter |
| 03099 | hypothetical protein |
| 03122 | peroxidase |
| 03143 | phosphotransferase family protein |
| 03144 | hypothetical protein |
| 03145 | hypothetical protein |
| 03146 | macrolide glycosyltransferase |
| 03147 | spermidine/putrescine ABC transporter, ATP-binding protein |
| 03150 | spermidine/putrescine-binding periplasmic protein |
| 03151 | GntR family transcriptional regulator |
| 03152 | glycosyl transferase family protein |
| 03153 | FAD dependent oxidoreductase |
| 03167 | hypothetical protein |
| 03168 | ATP-dependent DNA helicase |
| 03171 | succinylglutamate desuccinylase/aspartoacylase |
| 03172 | hypothetical protein |
| 03237 | glutathione S-transferase |
| 03320 | hypothetical protein |
| 03329 | alpha/beta hydrolase fold protein |
| 03330 | LysR family transcriptional regulator |
| 03331 | demethylmenaquinone methyltransferase |
| 03333 | dimethylmenaquinone methyltransferase |
| 03345 | hypothetical protein |
| 03378 | LuxR family transcriptional regulator |
| 03540 | glycerol-3-phosphate cytidyltransferase |
| 03541 | polysaccharide biosynthesis protein |
| 03544 | glucosyltransferase |
| 03545 | glycosyltransferase |
| 03584 | hypothetical protein |
| 03593 | hypothetical protein |
| 03633 | surface presentation of antigens protein |
| 04102 | hypothetical protein |
| 04148 | two component LuxR family transcriptional regulator |
| 04150 | dihydrolipoamide acetyltransferase |
| 04187 | hypothetical protein |
| 04190 | histidine kinase |
| 04690 | GntR family transcriptional regulator |
| 04692 | hypothetical protein |
