# Supplementary material for: The Complete Genome Sequence of the Plant Growth-Promoting Bacterium Pseudomonas sp. UW4
Source: PLoS One. 2013 Mar 13;8(3):e58640. doi: 10.1371/journal.pone.0058640 (PMC3596284; doi:10.1371/journal.pone.0058640)
Supplement: Table S15 — GenBank accession numbers of the sequences used in the analysis of UW4 taxonomy. (DOCX) [file pone.0058640.s018.docx]

Table S15. GenBank Accession Numbers of the Sequences Used in the Analysis of UW4 Taxonomy.

| Species | 16S rRNA | | *gyrB* | *rpoD* | *rpoB* |
| --- | --- | --- | --- | --- | --- |
| *P. abietaniphila* | AJ011504 | FN554166 | | FN554447 | AJ717416 |
| *P. aeruginosa* | AF094713 | AJ633104 | | AJ633568 | AJ717442 |
| *P. agarici* | AJ308298 | AB039456 | | AB039560 | AJ717477 |
| *P. alcaligenes* | AB680567 | AB039388 | | AB039606 | AJ748191 |
| *P. alcaliphila* | AB030583 | FN554167 | | FN554448 | AJ717463 |
| *P. amygdali* | NR_036999 | AB039474 | | AB039510 | AJ717462 |
| *P. anguilliseptica* | AB021376 | FN554168 | | FN554449 | FN554726 |
| *P. antarctica* | HE586386 | FN554169 | | FN554450 | FN554727 |
| *P. argentinensis* | AY691188 | FN554170 | | FN554451 | FN554728 |
| *P. asplenii* | AB021397 | AB039473 | | AB039594 | AJ717432 |
| *P. avellanae* | AJ889838 | FN554173 | | FN554454 | AJ717469 |
| *P. azotifigens* | AB189452 | FN554174 | | FN554455 | FN554729 |
| *P. azotoformans* | PSEIAM08 | AB039411 | | AB039547 | AJ717458 |
| *P. balearica* | AF054936 | AB039394 | | AJ633565 | AJ717480 |
| *P. borbori* | AM114527 | FN554175 | | FN554456 | FN554730 |
| *P. brassicacearum* | AJ292381 | AM084675 | | AM084334 | AJ717436 |
| *P. brenneri* | AF268968 | FN554176 | | FN554457 | AJ717482 |
| *P. cannabina* | AJ492827 | FN554177 | | FN554458 | AJ717453 |
| *P. caricapapayae* | PSEATCC09 | AB039454 | | AB039507 | AJ717437 |
| *P. cedrina* | AF064461 | FN554178 | | FN554459 | AJ717424 |
| *P. chlororaphis* | FJ652610 | FJ652718 | | D86036 | AJ717478 |
| *P. aurantiaca* | AB021412 | FN554171 | | FN554452 | AJ717426 |
| *P. aureofaciens* | AJ308300 | FN554172 | | FN554453 | AJ717421 |
| *P. cichorii* | AB021398 | AB039436 | | AB039524 | AJ717418 |
| *P. citronellolis* | AB021396 | AB039452 | | AB039604 | FN568270 |
| *P. congelans* | AJ492828 | FN554179 | | FN554460 | FN554731 |
| *P. corrugata* | AF348508 | AB039466 | | AB039574 | AJ748175 |
| *P. costantinii* | AF374472 | FN554180 | | FN554461 | FN554732 |
| *P. cremoricolorata* | AB060137 | FN554181 | | FN554462 | AJ717476 |
| *P. extremorientalis* | AF405328 | FN554182 | | FN554464 | FN554733 |
| *P. ficuserectae* | AB021378 | AB039418 | | AB039501 | AJ717457 |
| *P. flavescens* | AJ308320 | FN554183 | | FN554465 | AJ717468 |
| *P. fluorescens* | AF094725 | AB039492 | | AB039624 | HE586409 |
| *P. fragi* | AF094733 | FN554184 | | FN554466 | AJ717444 |
| *P. frederiksbergensis* | FR750403 | AM084676 | | AM084335 | AJ786298 |
| *P. fulva* | AB046998 | AB039440 | | AB039599 | AJ717419 |
| *P. fuscovaginae* | AB021381 | FN554185 | | FN554467 | AJ717433 |
| *P. gessardii* | AF074384 | FN554186 | | FN554468 | AJ717438 |
| *P. graminis* | NR_026395 | FN554187 | | FN554469 | AJ717429 |
| *P. grimontii* | AF268029 | FN554188 | | FN554470 | AJ717439 |
| *P. guineae* | AM491810 | FN554189 | | FN554471 | FN554734 |
| *P. indica* | AB681925 | FN554190 | | FN554472 | AJ717481 |
| *P. jessenii* | NR_024918 | FN554191 | | FN554473 | AJ717447 |
| *P. jinjuensis* | AB681927 | FN554192 | | FN554474 | FN554735 |
| *P. kilonensis* | AJ292426 | AM084677 | | AM084336 | AJ717472 |
| *P. knackmussii* | NR_041702 | FN554193 | | FN554475 | FN554736 |
| *P. koreensis* | AB495129 | FN554194 | | FN554476 | FN554737 |
| *P. libanensis* | NR_024901 | FN554195 | | FN554477 | AJ717454 |
| *P. lini* | AB649011 | FN554196 | | FN554478 | AJ717466 |
| *P. lundensis* | AB021395 | FN554197 | | FN554479 | AJ717428 |
| *P. lutea* | NR_029103 | FN554198 | | FN554480 | FN554738 |
| *P. luteola* | AJ871471 | FN554199 | | FN554481 | AJ717452 |
| *P. mandelii* | AF058286 | FN554200 | | FN554482 | AJ717435 |
| *P. marginalis* | AB021401 | AB039472 | | AB039578 | AJ717425 |
| *P. marincola* | AB301071 | FN554201 | | FN554483 | FN554739 |
| *P. mediterranea* | JQ904748 | AM084678 | | AM084337 | AJ717449 |
| *P. meliae* | AB021382 | FN554202 | | FN554484 | AJ717486 |
| *P. mendocina* | AJ308310 | AJ633103 | | AJ633567 | AJ748169 |
| *P. meridiana* | AJ537602 | FN554203 | | FN554485 | FN554740 |
| *P. migulae* | NR_024927 | FN554204 | | FN554486 | AJ717446 |
| *P. mohnii* | AM293567 | AM293561 | | FN554487 | FN554741 |
| *P. monteilii* | AF064458 | FN554205 | | FN554488 | AJ717455 |
| *P. moorei* | AM293566 | AM293560 | | FN554489 | FN554742 |
| *P. moraviensis* | AY970952 | FN554206 | | FN554490 | FN554743 |
| *P. mosselii* | AF072688 | FN554207 | | FN554491 | FN554744 |
| *P. mucidolens* | PSEIAM16 | AB039409 | | AB039546 | AJ717427 |
| *P. nitroreducens* | AM088473 | FN554208 | | FN554492 | AJ717448 |
| *P. oleovorans* | AB680450 | AB039396 | | AB039601 | AJ717461 |
| *P. orientalis* | AF064457 | FN554209 | | FN554493 | AJ717434 |
| *P. oryzihabitans* | GQ250598 | FN554210 | | FN554494 | AJ717470 |
| *P. otitidis* | NR_043289 | FN554211 | | FN554495 | FN554745 |
| *P. pachastrellae* | AB125366 | FN554212 | | FN554496 | FN554746 |
| *P. palleroniana* | AY091527 | FN554213 | | FN554497 | FN554747 |
| *P. panacis* | AY787208 | FN554214 | | FN554498 | FN554748 |
| *P. panipatensis* | EF424401 | FN554215 | | FN554499 | FN554749 |
| *P. parafulva* | AB060132 | FN554216 | | FN554500 | AJ717471 |
| *P. peli* | AM114534 | FN554217 | | FN554501 | FN554750 |
| *P. pertucinogena* | AB021380 | DQ350613 | | FN554502 | AJ717441 |
| *P. plecoglossicida* | AB009457 | FN554218 | | FN554503 | AJ717456 |
| *P. poae* | AJ492829 | FN554219 | | FN554504 | FN554751 |
| *P. proteolytica* | AJ537603 | FN554220 | | FN554505 | FN554752 |
| *P. pseudoalcaligenes* | AB021379 | AB039439 | | AB039598 | AJ717430 |
| *P. psychrophila* | NR_028619 | FN554221 | | FN554506 | AJ717464 |
| *P. psychrotolerans* | NR_042191 | FN554222 | | FN554507 | FN554753 |
| *P. putida* | AF094736 | AB039451 | | AB039581 | AJ864841 |
| *P. reinekei* | AM293565 | AM293559 | | FN554508 | FN554754 |
| *P. resinovorans* | AB021373 | FN554223 | | FN554509 | AJ717479 |
| *P. rhizosphaerae* | AY152673 | FN554224 | | FN554510 | FN554755 |
| *P. rhodesiae* | NR_024911 | FN554225 | | FN554511 | AJ717431 |
| *P. salomonii* | AY091528 | FN554226 | | FN554512 | FN554756 |
| *P. savastanoi* | DQ318862 | AB039468 | | AB039513 | AJ717422 |
| *P. simiae* | AJ936933 | FN554227 | | FN554513 | FN554757 |
| *P. straminea* | NR_036908 | AB039410 | | AB039600 | FN554758 |
| *P. stutzeri* | AF094748 | AB039392 | | AB039613 | AJ748189 |
| *P. synxantha* | PSEIAM24 | AB039415 | | AB039550 | AJ717420 |
| *P. syringae* | AJ308316 | AB039428 | | AB039516 | FN554759 |
| *P. taetrolens* | PSEIAM26 | AB039412 | | AB039523 | AJ717423 |
| *P. thermotolerans* | AJ311980 | FN554228 | | FN554514 | FN554760 |
| *P. thivervalensis* | AF100323 | AM084679 | | AM084338 | AM084680 |
| *P. tolaasii* | AF348507 | AB039423 | | AB039561 | AJ748160 |
| *P. tremae* | AJ492826 | FN554229 | | FN554463 | FN554761 |
| *P. trivialis* | AJ492831 | FN554230 | | FN554515 | FN554762 |
| *P. umsongensis* | NR_025227 | FN554231 | | FN554516 | FN554763 |
| *P. vancouverensis* | AJ011507 | FN554232 | | FN554517 | AJ717473 |
| *P. veronii* | AB021411 | FN554233 | | FN554518 | AJ717445 |
| *P. viridiflava* | AF094751 | AB039427 | | AB039520 | FN554764 |
| *P. xanthomarina* | AB176954 | AM905836 | | AM905872 | FN554765 |
